# Significant impact of time-of-day variation on metformin pharmacokinetics

**DOI:** 10.1007/s00125-023-05898-4

**Published:** 2023-03-17

**Authors:** Denise Türk, Nina Scherer, Dominik Selzer, Christiane Dings, Nina Hanke, Robert Dallmann, Matthias Schwab, Peter Timmins, Valerie Nock, Thorsten Lehr

**Affiliations:** 1 Clinical Pharmacy, Saarland University, Saarbrücken, Germany; 2 Division of Biomedical Sciences, Warwick Medical School, University of Warwick, Coventry, UK; 3 Dr. Margarete Fischer-Bosch-Institute of Clinical Pharmacology, Stuttgart, Germany; 4 Departments of Clinical Pharmacology, Pharmacy and Biochemistry, University of Tübingen, Tübingen, Germany; 5 Cluster of Excellence iFIT (EXC2180) ‘Image-guided and Functionally Instructed Tumor Therapies’, University of Tübingen, Tübingen, Germany; 6 Department of Pharmacy, University of Huddersfield, Huddersfield, UK; 7 Boehringer Ingelheim Pharma GmbH & Co. KG, Biberach, Germany

**Keywords:** Chronopharmacology, Empirical modelling, Mechanistic modelling, Metformin, Pharmacokinetics, Renal excretion, Transporter

## Abstract

**Aims/hypothesis:**

The objective was to investigate if metformin pharmacokinetics is modulated by time-of-day in humans using empirical and mechanistic pharmacokinetic modelling techniques on a large clinical dataset. This study also aimed to generate and test hypotheses on the underlying mechanisms, including evidence for chronotype-dependent interindividual differences in metformin plasma and efficacy-related tissue concentrations.

**Methods:**

A large clinical dataset consisting of individual metformin plasma and urine measurements was analysed using a newly developed empirical pharmacokinetic model. Causes of daily variation of metformin pharmacokinetics and interindividual variability were further investigated by a literature-informed mechanistic modelling analysis.

**Results:**

A significant effect of time-of-day on metformin pharmacokinetics was found. Daily rhythms of gastrointestinal, hepatic and renal processes are described in the literature, possibly affecting drug pharmacokinetics. Observed metformin plasma levels were best described by a combination of a rhythm in GFR, renal plasma flow (RPF) and organic cation transporter (OCT) 2 activity. Furthermore, the large interindividual differences in measured metformin concentrations were best explained by individual chronotypes affecting metformin clearance, with impact on plasma and tissue concentrations that may have implications for metformin efficacy.

**Conclusions/interpretation:**

Metformin’s pharmacology significantly depends on time-of-day in humans, determined with the help of empirical and mechanistic pharmacokinetic modelling, and rhythmic GFR, RPF and OCT2 were found to govern intraday variation. Interindividual variation was found to be partly dependent on individual chronotype, suggesting diurnal preference as an interesting, but so-far underappreciated, topic with regard to future personalised chronomodulated therapy in people with type 2 diabetes.

**Graphical abstract:**

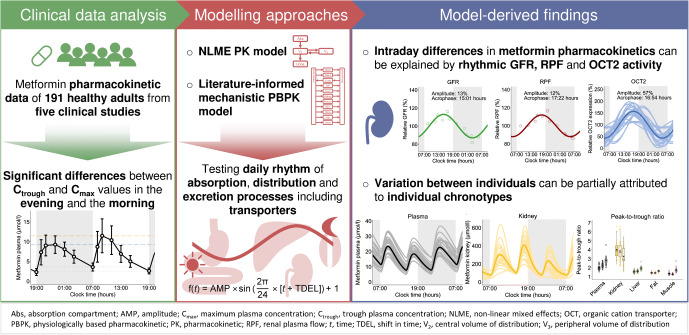

**Supplementary Information:**

The online version contains peer-reviewed but unedited supplementary material available at 10.1007/s00125-023-05898-4.

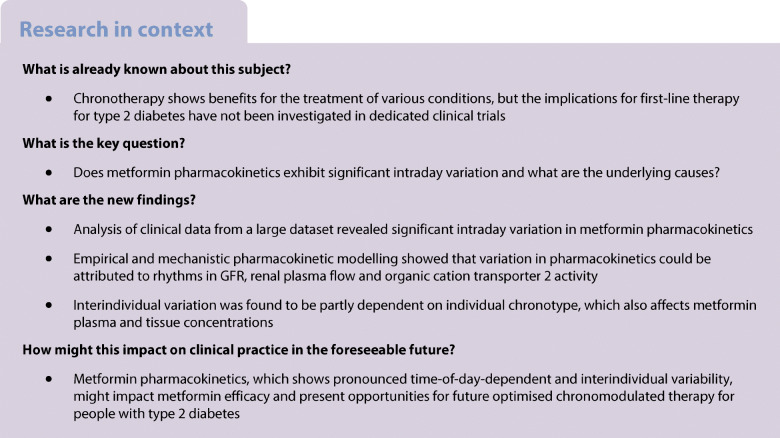



## Introduction

Metformin is recommended as first-line therapy for type 2 diabetes [1] and predominantly acts in the gastrointestinal system by decreasing glucose uptake from the lumen and increasing glucagon-like peptide-1 secretion [2]. Furthermore, it leads to inhibition of hepatic gluconeogenesis [3] and increased insulin-stimulated glucose uptake into other organs e.g. skeletal muscle [4], resulting in a reduction in blood glucose levels. Recent work suggests that metformin therapy is associated with a preventive effect against cancer and could even be a useful adjuvant in cancer therapy [5].

Metformin is highly soluble, exhibits a low permeability and retains a positive charge over the whole range of physiological pH. Hence, its absorption, distribution and excretion strongly depend on active transport processes to cross biological membranes. Incomplete transporter-mediated absorption from the upper intestine yields a moderate bioavailability of about 55% [6]. Pharmacokinetic metformin data indicate high inter- and intraindividual variation [6]. Metformin is not metabolised and is mainly excreted in urine passively via glomerular filtration and actively by consecutive action of organic cation transporter (OCT) 2 and multidrug and toxin extrusion proteins (MATEs) [6].

While the pharmacokinetics of metformin is generally well understood, the influence of time-of-day on metformin pharmacology, in particular, has not yet been described. Analysing plasma concentration–time profiles of a twice daily metformin administration from a study conducted with the intention of investigating bioequivalence of different metformin formulations [7] revealed similar mean AUC values during day and night. However, altered plasma curve shapes as well as sizeable time-of-day variations of trough plasma concentrations (C_trough_) and maximum plasma concentrations (C_max_) were found. Many body functions, like the GFR and other excretion processes as well as absorption and metabolic processes, underlie intraday variations, resulting in changes of drug exposure and, subsequently, in daily rhythms of efficacy or toxicity [8]. Thus, observed variability in metformin plasma concentrations might be explained by time-of-day dependent pharmacokinetics. To date, however, no dedicated analyses were available in published literature to assess time-dependent alteration of metformin pharmacology in humans.

In general, large interindividual differences in diurnal preference (also referred to as ‘chronotype’) have been observed, for example as preferred wake-up and sleep times [9]. Chronotherapy, i.e. taking daytime into account for drug administration, might have clinical benefits in various indications [10–12]. Considering the individual chronotype within the context of personalised precision chronotherapy may improve therapy outcomes, as proposed as a potential treatment option for cancer patients [13].

Here, we (1) investigated whether metformin pharmacokinetics in humans exhibits a significant intraindividual difference depending on time-of-day of administration; (2) quantified the magnitude of the effect using non-linear mixed effects (NLME) pharmacokinetic modelling on a large clinical dataset of individual metformin concentration measurements; (3) generated hypotheses regarding sources of such daily variation and tested the underlying mechanisms using a literature-informed physiologically based pharmacokinetic (PBPK) modelling approach; (4) partly explained interindividual variability based on the model-determined chronotype; and (5) simulated the daily rhythms of metformin concentrations in relevant tissues, which could support the assessment of clinical relevance in future work.

## Methods

### Clinical dataset

Individual metformin measurements from five clinical studies were used. Metformin was administered as immediate- (IR) and extended-release (ER) formulations of 500–2000 mg once to three times daily in single and multiple day regimens. All studies have been approved by the local ethics committees and informed consent was obtained from all participants before study entry. Results from studies II–V have not previously been published. Detailed information on all studies, including number and demographics of participants, inclusion and exclusion criteria and exact dosing/sampling schedules are provided in electronic supplementary material (ESM) Tables 1–5 and ESM Figs 1–5.

### Statistical analysis

Individual plasma measurements were analysed separately for differences between C_trough_ values measured immediately before the next dose in the morning (‘C_trough,morning_’) and the evening (‘C_trough,evening_’) as well as C_max_ values measured after the morning dose (‘C_max,morning_’) and the evening dose (‘C_max,evening_’). Details on statistical analysis are provided in ESM Section 1.2.

### NLME pharmacokinetic modelling

An empirical pharmacokinetic model of metformin was implemented in NONMEM (version 7.4.3, ICON Development Solutions, Ellicott City, MD, USA), informed by individual data using NLME techniques. The final model was built and evaluated in a three-step procedure by: (1) developing a structural model by checking one-, two- and three-compartment disposition models as well as zero-order, first-order and Michaelis–Menten absorption and elimination kinetics; (2) quantifying interindividual and residual variabilities based on the structural model by testing variability on each model parameter; and (3) investigating the effects of the covariates (e.g. sex, age, body weight, serum creatinine, administered dose, formulation, comedication and food intake) using a forward inclusion and backward elimination procedure with significance levels of 5% and 0.1%, respectively.

A function assuming sinusoidal oscillations with a 24 h period (Equation 1) was tested on respective model parameters to identify whether a daily rhythm of metformin absorption, distribution and/or excretion processes improves the description of metformin plasma and urine concentrations:

1 $$ \mathrm{f}(t)=\mathrm{AMP}\times \sin \left(\frac{2\uppi}{24}\times \left[t+\mathrm{TDEL}\right]\right)+1 $$

where *t* = time, AMP = amplitude and TDEL = shift in time. Values for amplitude and shift were optimised by fitting model simulations to observed metformin profiles.

Details on final model selection and evaluation are provided in ESM Section 1.3.

### Literature-informed mechanistic PBPK modelling

The literature was extensively searched for physiological conditions linked to rhythmicity in absorption, distribution and excretion of metformin, including metformin-specific transporters.

To elucidate the key variables with impact on metformin pharmacokinetics, a mechanistic whole-body PBPK modelling approach was applied, where organs are represented by compartments that are connected via blood flow. The change of drug concentration in these compartments over time is described by differential equations. Mechanistic implementation of transport processes at their respective sites of action allows simulation and prediction of drug concentrations in all relevant organs and body sites. A published whole-body PBPK model of metformin [14] developed via the Open Systems Pharmacology Suite (version 8.0, https://www.open-systems-pharmacology.org/) using metformin studies in healthy volunteers after intravenous and oral administration in fasted and fed state (single and multiple-dose, dosing range 0.001–2550 mg) was used as a basis for further investigation. The model includes active transport by plasma membrane monoamine transporter (PMAT), OCT1 as well as consecutive action of OCT2 and MATE1.

Time-of-day variation of pharmacokinetic-related processes and physiological conditions identified in the literature was tested with the PBPK model. By modulating relevant model parameter values over time with an oscillation function (Equation 1), the influence of each process on metformin pharmacokinetics was tested separately. Amplitude and acrophase (i.e. clock time of maximal activity) of the tested rhythmic processes were implemented as reported previously or optimised by fitting simulations to observed aggregated metformin plasma concentration–time profiles from study I. The impact of each tested process on metformin pharmacokinetics was evaluated visually and quantitatively by calculating mean relative deviations (MRDs) and geometric mean fold errors (GMFEs) (ESM Equations 1 and 2), to estimate the model accuracy for metformin concentration–time profiles as well as C_trough_ and C_max_ ratios. Details on model extension and performance evaluation are provided in ESM Section 1.4. Information about expression of relevant transport proteins is presented in ESM Table 6.

## Results

### Clinical dataset

The dataset derived from five studies included data on 191 healthy adults (65% men, 18–50 years) with 7476 plasma and 316 urine levels of metformin. Of these, 21.4% of plasma and 100% of urine measurements were observed after administration of IR formulations. Pharmacokinetic profiles covering at least one dosing interval were available for all individuals, with additional C_trough_ measurements for multiple-dose administration studies (studies I and II: 1000 mg twice daily, and study III: 850 mg three times daily) that allowed further investigations of intraday variation in pharmacokinetics.

### Statistical analysis

Plasma concentration–time profiles from studies I and II were used for the investigation of differences in individual mean C_trough_,_morning_ and C_trough,evening_ values. Statistical analyses revealed 42% higher mean C_trough_ measurements in the morning compared with the evening (*p*=0.00016). Moreover, individual C_trough_ measurements exhibited a large intraindividual variability of up to 75%. Linear mixed model analysis that included all individual C_trough_ measurements also confirmed significantly higher C_trough_ measurements in the morning (*p*<0.0001).

**Fig. 1:**
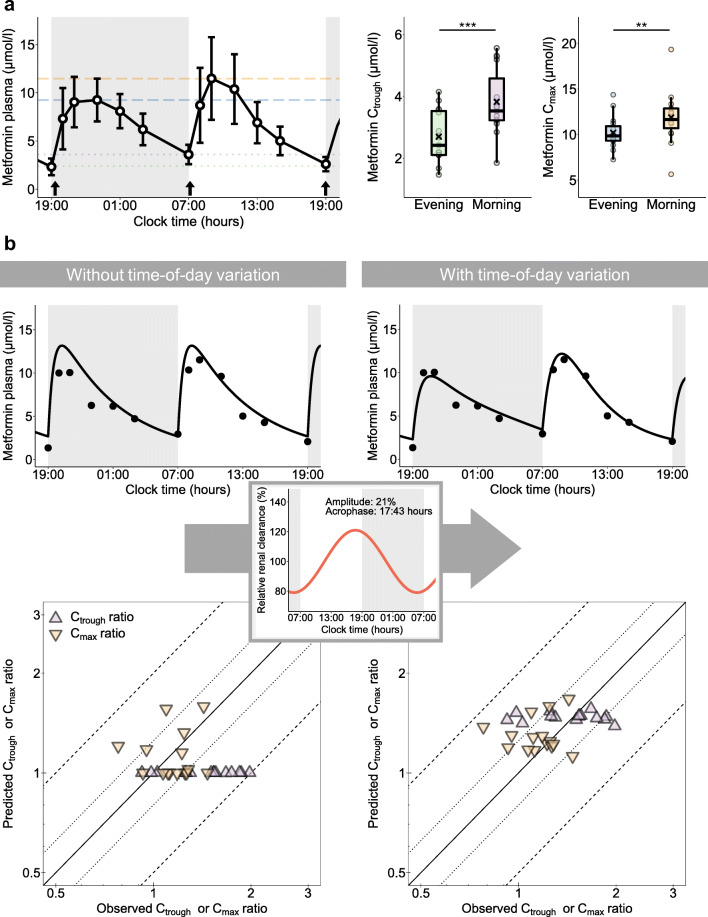
Investigation of daytime-dependent metformin pharmacokinetics with concentration measurements from study I [7]. (**a**) Statistically significant differences between trough plasma concentrations (C_trough_) measured in the morning compared with the evening and maximum plasma concentrations (C_max_) measured in the morning compared with the evening were found. Data are shown as arithmetic means ± SD. Metformin administration (1000 mg twice daily) is indicated by arrows. Grey areas indicate night-time. In the box plots, mean C_trough_ and C_max_ values are indicated by crosses, individual values (*n*=15) by dots. Boxes represent the distance between first and third quartiles (IQR). Whiskers range from smallest to highest value (<1.5 × IQR). **p<0.01; ***p<0.001. (**b**) Performance of the NLME model without and with time-of-day variation via the estimated oscillation function (insert and Equation 1) applied on clearance. Representative individual plasma concentration–time profiles (*n*=1) are plotted after twice daily administration of 1000 mg metformin. Dots indicate observed data and lines indicate model predictions. Goodness-of-fit plots show comparisons of all predicted and observed individual C_trough_ and C_max_ ratios after twice daily administration of 1000 mg metformin. The straight solid line marks the line of identity, dotted lines indicate 1.25-fold and dashed lines indicate twofold deviations

Although differences were less pronounced for C_max_, i.e. 16% higher mean C_max_ values in the morning compared with the evening, measurements were significantly different with *p*=0.0053 for *t* test analysis of mean values. Furthermore, for C_max_, large intraindividual variability was observed, with variability up to 52%. In the mixed model analysis, the findings from analysing the means could be confirmed with *p*=0.0063. A summary of the statistical analysis is shown in Fig. 1a and ESM Figs 6 and 7.

### NLME pharmacokinetic modelling

All individual plasma and urine measurements from studies I–V were used for model development and were best described by a two-compartment disposition model with first-order absorption, distribution and clearance. IR formulations were modelled via first-order absorption and ER formulations by a zero-order release preceded the first-order absorption. Interindividual variability could be identified for clearance, central volume of distribution and bioavailability. Implementation of food intake, formulation and dose as significant covariates reduced the interindividual variabilities for clearance, volume of distribution and bioavailability by 14%, 75% and 52%, respectively. Administration after food intake led to a 1.9-fold higher relative bioavailability and a 0.6-fold slower absorption rate constant, but a 5.1-fold increased release duration for the ER formulation. The bioavailability of the ER formulation was 1.1-fold higher compared with the IR formulation. The metformin dose was implemented as a covariate using an exponential function (ESM Equation 3), leading to a decreased relative bioavailability for higher administered doses of metformin.

Daily variation was tested for absorption, distribution as well as clearance parameters. Model performance significantly improved if a daily rhythm on metformin clearance was incorporated (*p*<1.0 × 10^–100^), applying an estimated amplitude of 21% and an acrophase at 17:43 hours. Parameter estimates of the model are provided in ESM Table 7, and the model structure is presented in ESM Fig. 8. The performance of the NLME model without and with daily rhythm is presented with plasma concentration–time profiles and goodness-of-fit plots in Fig. 1b and ESM Figs 9–13, indicating good performance of the model including daily variation, with 95% and 83% of predicted individual metformin plasma and urine concentrations, respectively, within twofold of the observed values.

C_trough_ and C_max_ predictions showed smaller errors for the model with rhythmic renal clearance compared with the model assuming constant renal clearance, quantified by a decrease of mean GMFEs from 1.45 to 1.21 for C_trough_ and 1.21 to 1.19 for C_max_ ratios of study I (Fig. 1b and ESM Figs 14 and 15). Comparison of conditional weighted residuals vs time and predicted concentration is presented in ESM Figs 16 and 17. Further details on modelling results are provided in ESM Section 2.2.

### Literature-informed mechanistic PBPK modelling

Previous studies reported daily rhythm in absorption- and distribution-related physiological conditions, namely gastric pH, gastric emptying time, gut motility, blood flow to the gastrointestinal tract and hepatic blood flow, with effects on drug solubility, bioavailability, transit time through the gastrointestinal tract and distribution in the body. For excretion-related processes, rhythmic GFR and renal blood flow have been described (ESM Table 8). In addition, daily variation of active transport processes in the liver and kidney have been observed [8]. No rhythm was reported for PMAT (*SLC29A4*), mainly involved in intestinal absorption of metformin, either in humans or in animals. For OCT1 (*SLC22A1*), the transporter mainly responsible for metformin uptake into hepatocytes, no human time-series data were available. However, in mice, hepatic *Slc22a1* mRNA expression is not rhythmic [15]. Regarding renal transporters, *Slc47a1* (MATE1) is not rhythmic [15, 16], while for *Slc22a2* (OCT2) expression, one study reported significant daily rhythms [17]. Again, no human expression data were available to investigate *SLC22A2* (OCT2) rhythmicity.

**Fig. 2:**
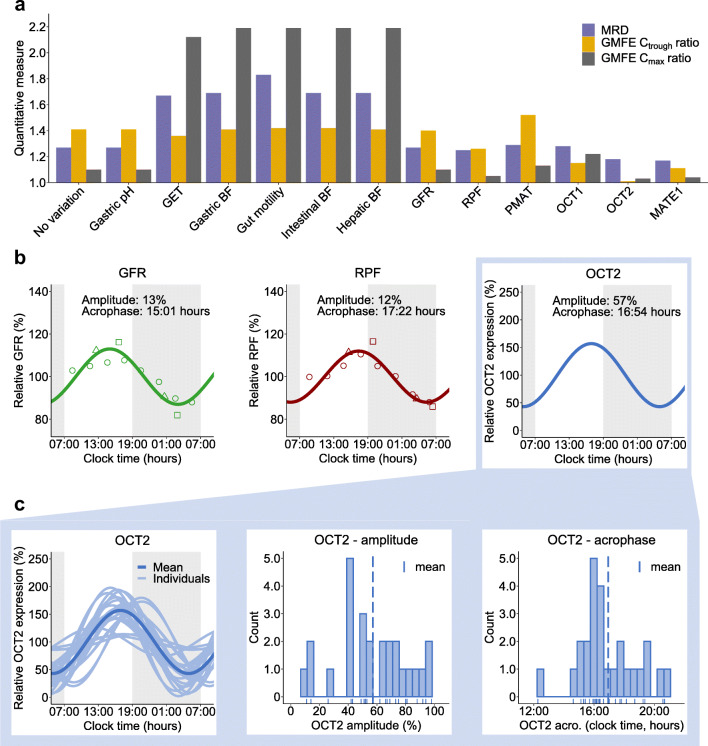
Implementation of a daily rhythm in the metformin PBPK model. (**a**) Hypothesis testing. Rhythmic physiological processes and transporter activities tested using the PBPK model with the respective prediction performance metrics, i.e. MRDs and GMFEs. (**b**, **c**) Final PBPK model processes with rhythmic excretion. (**b**) Time-of-day variation of GFR and RPF as reported in the literature [18–20] (measurements from different reports indicated by dots, triangles and squares) and OCT2 implemented in the final PBPK model. (**c**) Rhythm of OCT2 was optimised with the PBPK model for each individual, and individual OCT2 parametrisation is shown as distribution of individually optimised OCT2 amplitudes and acrophases (*n*=26). acro, acrophase; BF, blood flow; GET, gastric emptying time

These potential factors introducing time-of-day variation were tested in the PBPK model to confirm and explain findings from the NLME model regarding observed time-of-day variation in metformin pharmacokinetics. Temporal variations of gastrointestinal and distribution-related processes as well as GFR and renal plasma flow (RPF) were modelled with literature values for amplitudes and acrophases, expressing amplitudes from 12–56% (ESM Table 8). However, judging by GMFEs for C_trough_ and C_max_ ratios, rhythmic absorption-, distribution- and passive excretion-related processes on metformin pharmacokinetics did not lead to an improved description of metformin concentration–time profiles, especially not for both C_trough_ and C_max_ values compared with the base PBPK model without any temporal variation. The daily variation of transport rates was modelled with optimised values for amplitudes and acrophases. Introducing oscillation in PMAT and OCT1 activities resulted in insufficient descriptions of both metformin C_trough_ and C_max_ ratios, while time-of-day variation in activity of excretion-related transporters, i.e. OCT2 and MATE1, improved descriptions of observed plasma concentrations as well as C_trough_ and C_max_ ratios (Fig. 2a and ESM Figs 18 and 19 under Section 2.3.2). In the final model, metformin plasma concentration–time profiles were modelled incorporating a combination of a daily rhythm in GFR, renal blood flow and OCT2 (Fig. 2b), which is consistent with published findings for human renal physiology [17–20]. PBPK model parameters are listed in ESM Tables 9 and 10 under Section 2.3.3.

### Individual differences in the daily rhythms of metformin pharmacokinetics

**Fig. 3:**
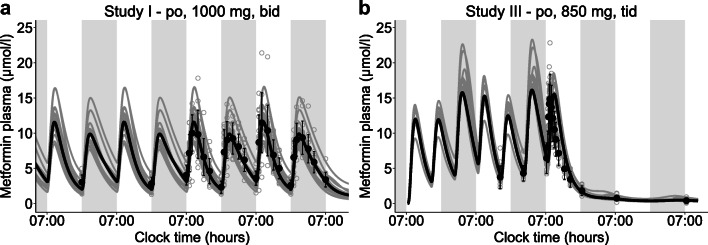
Mean (black lines) and individual (grey lines) PBPK model predictions of metformin plasma concentration–time profiles compared with measurements from (**a**) study I (*n*=15) and (**b**) study III (*n*=11) [7, 39]. Closed black dots indicate arithmetic means ± SD, open grey dots indicate individual measurements. Grey areas indicate night-time. bid, twice daily; po, oral; tid, three times daily

Individual metformin profiles exhibited a large interindividual variability and were insufficiently described using the same rhythmic OCT2 parametrisation for all participants. To account for interindividual variability in OCT2 baseline activity (caused by e.g. genetic polymorphisms), transport rate constant (*k*_cat_) values were optimised for individual profiles with mean *k*_cat_=57680 1/min (CV=69%; *n*=26). Individual chronotypes were estimated by calculating OCT2 amplitudes and acrophases separately for each individual with a mean amplitude of 57% (CV=43%) and mean acrophase at 16:54 hours (CV=39%). The distribution of individual amplitudes and acrophases is shown in Fig. 2c, while no correlation of OCT2 parameters has been determined (ESM Fig. 20). Predicted mean and individual plasma concentration–time profiles from studies I and III are shown in Fig. 3, agreeing with observed higher C_trough_ values before the morning dose and higher C_max_ values after the morning dose when administered every 12 h (Fig. 3a). Highest C_max_ values were predicted during the night for the three-times daily regimen (Fig. 3b). Predicted plasma concentration–time profiles for all studies compared with observed data are shown in ESM Figs 21–25. Goodness-of-fit plots demonstrate that 93% of predicted plasma concentration values from studies I and III lie within twofold of observed values (ESM Fig. 26). Comparisons of predicted and observed C_trough_ and C_max_ ratios are shown in ESM Fig. 27.

### Simulations in relevant tissues

**Fig. 4:**
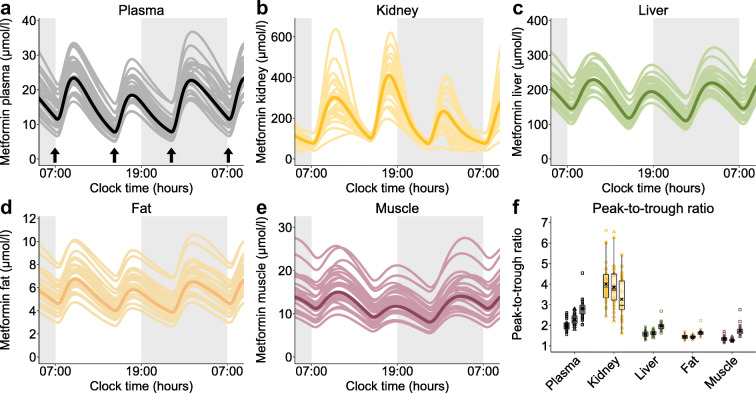
PBPK model simulations of plasma and tissue concentration–time profiles of an oral administration of three-times daily 1000 mg metformin (highest recommended dose according to the German prescribing information [21]) at 07:00, 15:00 and 23:00 hours (indicated by arrows). (**a**–**e**) Comparison of metformin levels in (**a**) plasma, (**b**) kidney tissue, (**c**) liver tissue, (**d**) fat tissue and (**e**) muscle tissue. Respective simulations with a mean parameter set of OCT2 *k*_cat_, amplitude and acrophase are shown as dark lines, simulations with individual parameter sets (*n*=26) are shown as light lines. Grey areas indicate night-time. (**f**) Comparison of metformin peak-to-trough ratios for simulations in plasma and tissues. The three box plots per tissue give peak-to-trough ratios after metformin administration at 07:00, 15:00 and 23:00 hours. Dots (peak 1), triangles (peak 2) and squares (peak 3) show individual peak-to-trough ratios (*n*=26), crosses indicate mean values. Boxes represent the distance between first and third quartiles (IQR). Whiskers range from smallest to highest value (<1.5 × IQR)

To investigate the impact of daily modulation of metformin pharmacology on its exposure in tissues, plasma, kidney, liver, fat and muscle tissue concentrations were simulated in steady-state, administering the highest recommended metformin dose of 1000 mg three times daily [21]. Simulated metformin concentration–time profiles for plasma and tissues showed substantial interindividual and intraday variability (Fig. 4a–e) and calculations of metformin peak-to-trough concentration ratios for each dosing interval within 1 day in plasma and tissues revealed intraday, intertissue and interindividual differences (Fig. 4f). As OCT2, the main contributor to metformin rhythm in the model, is expressed at the basolateral membrane of tubular epithelial cells, concentrations in the kidney showed an opposing trend due to a decreased transport of metformin into kidney cells at night, leading to increased concentrations in plasma and other tissues.

## Discussion

Chronotherapy might have a clinical benefit in various indications, discussed e.g. for therapy of cancer [10], rheumatoid arthritis [11] or metabolic diseases [12]. To date, the implications of chronopharmacology for treatment of type 2 diabetes have not been investigated in dedicated clinical trials. The presented work focused on statistical and in silico analyses of metformin pharmacokinetics covering interindividual variability with the help of NLME and PBPK modelling, revealing intraday changes in pharmacokinetics for metformin plasma and urine concentrations. Mechanistic mathematical modelling of metformin pharmacokinetics allowed the investigation of hypotheses for the underlying chrono-mechanisms and the integration of related findings in the overall context of diabetes therapy.

Statistical analyses showed significant differences between metformin C_trough_ as well as C_max_ values measured in the morning or the evening. To further investigate these differences and variations, empirical and mechanistic pharmacokinetic models were applied, as these methods have been proven useful to (1) test hypotheses and (2) investigate time-of-day dependence, e.g. for oral bioavailability and clearance of midazolam [22], light-triggered melatonin release [23] and heart rate [24].

The NLME pharmacokinetic modelling analysis led to an accurate description of metformin plasma concentrations, especially C_trough_ and C_max_ measurements, when a daily rhythm for systemic clearance was incorporated. In contrast, rhythms of other processes, e.g. the absorption, did not improve the modelling outcomes. The time-dependent effect on systemic clearance might be predominantly attributed to biologic rhythmic variations of kidney function, as metformin is exclusively eliminated renally. Rhythmic GFR and renal blood flow have been described [18–20] while the variation in GFR cannot be explained by the oscillation of renal blood flow alone [20]. In mice, the circadian clock in podocytes has been found to contribute to a rhythmic GFR [25], but the influence of further systemic factors are not completely understood [26]. However, the NLME estimated amplitude for the oscillation of metformin clearance was more pronounced compared with the published amplitude for GFR (21% vs 13%) [18–20], leading to the hypothesis of an additional daily rhythm in active secretion.

The PBPK modelling approach complemented the empirical NLME model, as it allowed the mechanistic implementation of individual physiology including demographics, kidney function as well as relevant transport proteins. Key processes from the NLME model were well in line with the independently developed PBPK model, and the additional rhythmic active secretion, proposed by analysing the NLME model outcomes, could be supported with the PBPK approach. Here, metformin plasma concentration–time profiles, especially C_max_ as well as C_trough_, were best described assuming a combination of rhythmic GFR, renal blood flow and tubular secretion rate. In the literature, there is no unambiguous evidence for rhythmic *SLC22A2* compared with *SLC47A1* (MATE1). Kidney expression data from mice presented by Zhang et al [16] reveal that *Slc47a1* is not rhythmic, similar to *Slc22a2*, but a slightly more pronounced rhythm in *Slc22a2* is shown. Oda et al [17] observed rhythmic *Slc22a2* but not *Slc47a1* expression profiles in nocturnal mice [17]. Baboons exhibit a rhythm in both *SLC22A2* and *SLC47A1* expression in the kidney medulla, but not in the kidney cortex, where the relevant proximate tubule cells are located [27]. Unfortunately, expression data of *SLC22A2* in human kidney cells are currently not available. However, due to the sequential action of OCT2 and MATEs in tubule cells, discrimination between both processes is challenging and dedicated studies are required to investigate the specific contributions of both OCT2 and MATEs. Model implementation of daily variation for both OCT2 and MATE1, also taking interindividual variability into account, was not feasible using the currently available data. If data from human kidneys become available, the model could be adjusted in future projects.

The influence of other possible rhythmic processes affecting drug pharmacokinetics, i.e. absorption- and distribution-related mechanisms, was quantified with a PBPK model, because these mechanisms might also affect metformin solubility, distribution and transit time through the gastrointestinal tract, bioavailability and tissue distribution [8]. Food intake has been reported to reduce metformin bioavailability of an IR formulation [28] but was not the main contributor to observed daily variations of metformin pharmacokinetics according to our PBPK model analysis. Only a minor contribution of daily rhythms in other absorption- and distribution-related processes has been assumed in previous work [15, 28–30]. This was confirmed, as a rhythmic absorption rate reduced NLME model performance, and PBPK modelling of rhythmic absorption- and distribution-related processes showed only a very small effect on plasma concentration–time profiles (ESM Figs 18 and 19). An altered metformin absorption, however, might have a more pronounced impact on its pharmacokinetics after administration of ER formulations, which was not addressed in this study due to the lack of clinical data.

Both the NLME and the PBPK models predict higher C_trough_ and C_max_ values of the morning dose compared with the evening dose after twice daily administration. The PBPK model predicts higher C_max_ values during the night when metformin is administered in a three-times daily regimen. This observation is expected, as maximum GFR, RPF and OCT2 activity are modelled in the late afternoon and minimum activity was assumed in the early morning. Hence, in comparison with the morning dose, higher metformin levels are expected after the night dose administered at 23:30 hours with increased C_trough_ values predicted in the morning due to lower elimination of metformin. Moreover, individual differences in night C_max_ compared with day C_max_ can be attributed to individual chronotypes estimated by the model. For the three-times daily regimen, frequent measurements during the night would be valuable to verify model hypotheses.

Since large interindividual differences in individual chronotypes have been observed [9], ‘chronotype’ like phase differences were hypothesised and an individual OCT2 parametrisation improved plasma concentration–time predictions. Whereas all data herein are from healthy volunteers, an extension to patients with diabetes or kidney failure might be challenging due to relevant pathophysiological changes. Moreover, rhythms in GFR, RPF and OCT2 could be different in patients compared with healthy individuals [31] and, thus, require further experimental data to adjust our model.

Both of our presented models assume active renal excretion as the main contributor to metformin pharmacokinetics. In our NLME model, a large interindividual variability (CV=68%) was estimated on the clearance processes. Other modelling work, e.g. by Stage and coworkers, identified less variability on the clearance (CV=25%) but found large interoccasion variability (up to CV=94%) on absorption and bioavailability processes [32]. Duong et al presented varying degrees of interindividual variability without modelling daytime variations but incorporated interoccasion variability as a random error term [33]. The comparison of parameter estimates from empirical NLME models is complex, as the models are non-mechanistic and were built for different purposes with different model structures and different datasets. It may be speculated that the modelled interoccasion variability might represent parts of the daytime variation, as the inclusion of daytime variation in our model reduced the interindividual variability on the central volume of distribution and the clearance significantly, by 36% and 11%, respectively.

Disruption of the circadian clock has been associated with the development of various diseases, such as metabolic and cardiovascular disorders [34]. Mistimed sleep, for example in shift workers, has been identified as a risk factor for developing type 2 diabetes [35], as this affects glucose tolerance and insulin sensitivity [36]. Additionally, a disrupted daily rhythm has been described in diabetes patients [37]. In diet-induced obese rats, targeting the disrupted clock using melatonin in combination with metformin led to an improved therapy outcome [38]. However, using chronobiological concepts to optimise the treatment of type 2 diabetes is not adopted in clinical practice yet. In mice, differences in the direct glucose-lowering effect of metformin and in blood lactic acid levels were observed if metformin was administered in the active or the rest phase of the animals [15], which would support the hypothesis of time-of-day dependent pharmacodynamics. An interesting future research question regarding clinical implication might focus on the extent to which intraday variation of metformin pharmacokinetics affects efficacy and toxicity in humans, i.e. the risk of lactic acidosis, which needs further investigation. Additionally, personalised chronotherapy might improve therapy outcomes for diabetes patients.

Statistical analyses as well as empirical and mechanistic pharmacokinetic modelling were successfully applied to generate and test hypotheses of the underlying chrono-mechanisms affecting metformin pharmacokinetics. Both modelling approaches suggest that rhythmic renal elimination had the strongest impact on metformin pharmacokinetics. Key variables of renal elimination were the rhythms in GFR, renal blood flow and OCT2-dependent transport rate. More broadly, our analyses demonstrated the strength of combining empirical and mechanistic pharmacokinetic modelling as a powerful toolchain to investigate scenarios with incomplete and missing clinical data. Furthermore, our results suggest a significant impact of chronotype on metformin pharmacology. Thus, this work might be a starting point for the translation of study results to therapy outcomes and risk assessment by individualised chronotherapy.

## Supplementary Information

ESM (PDF 8.81 MB)

## Data Availability

The datasets analysed during the current study from Bristol Myers Squibb (study I) and Boehringer Ingelheim (studies II—V) are not publicly available. Data from Bristol Myers Squibb are however available from the authors upon reasonable request and with the permission of Bristol Myers Squibb. Regarding the data from Boehringer Ingelheim, interested researchers are invited to consult the external research platform Vivli (https://vivli.org/) to request access to anonymised data.

## Abbreviations

C_max_: Maximum plasma concentration
C_trough_: Trough plasma concentration
ER: Extended-release
GMFE: Geometric mean fold error
IR: Immediate-release
*k*_cat_: Transport rate constant
MATE: Multidrug and toxin extrusion protein
MRD: Mean relative deviation
NLME: Non-linear mixed effects
OCT: Organic cation transporter
PBPK: Physiologically based pharmacokinetic
PMAT: Plasma membrane monoamine transporter
RPF: Renal plasma flow
