# Supplementary material for: Significant impact of time-of-day variation on metformin pharmacokinetics
Source: Diabetologia. 2023 Mar 17;66(6):1024–34. doi: 10.1007/s00125-023-05898-4 (PMC10163090; doi:10.1007/s00125-023-05898-4)
Supplement: Supplementary file 1 — (PDF 8.81 MB) [file 125_2023_5898_MOESM1_ESM.pdf]

Diabetologia

Significant impact of time-of-day variation on metformin pharmacokinetics

Electronic supplementary material (ESM)

Denise Türk[1], Nina Scherer[1], Dominik Selzer[1], Christiane Dings[1], Nina Hanke[1], Robert Dallmann[2], Matthias Schwab[3,4,5], Peter Timmins[6], Valerie Nock[7] and Thorsten Lehr[1]

Denise Türk and Nina Scherer contributed equally to this study.

[1] Clinical Pharmacy, Saarland University, Saarbrücken, Germany

[2] Division of Biomedical Sciences, Warwick Medical School, University of Warwick, Coventry, UK

[3] Dr. Margarete Fischer-Bosch-Institute of Clinical Pharmacology, Stuttgart, Germany

[4] Departments of Clinical Pharmacology, Pharmacy and Biochemistry, University of Tübingen, Tübingen, Germany

[5] Cluster of Excellence iFIT (EXC2180) 'Image-guided and Functionally Instructed Tumor Therapies', University of Tübingen, Tübingen, Germany

[6] Department of Pharmacy, University of Huddersfield, Huddersfield, UK

[7] Boehringer Ingelheim Pharma GmbH & Co. KG, Biberach, Germany

Corresponding Author:

Thorsten Lehr

Email: thorsten.lehr@mx.uni-saarland.de

ORCID of the author(s): Denise Türk: orcid.org/0000-0002-4047-402X, Robert Dallmann: orcid.org/0000-0002-7490-0218, Peter Timmins: orcid.org/0000-0002-5840-0678, Thorsten Lehr: orcid.org/0000-0002-8372-1465

Funding: MS was supported in part by the Robert Bosch Stiftung, Stuttgart, Germany, and the Deutsche Forschungsgemeinschaft (DFG, German Research Foundation) under Germany's Excellence Strategy - EXC 2180 – 390900677. TL received funding from Boehringer Ingelheim Pharma GmbH & Co. KG for personnel costs of NS, CD and NH. Authors' relationships and activities: NH is now an employee of Boehringer Ingelheim Pharma GmbH & Co. KG. VN is an employee of Boehringer Ingelheim Pharma GmbH & Co. KG. DT, NS, DS, CD, RD, MS, PT and TL declare that there are no relationships or activities that might bias, or be perceived to bias, their work.

1 ESM Methods

1.1 Clinical dataset

ESM Table 1. Information on study I (metformin bioavailability study by Timmins et al. [1])

**Study number**	**I**
Title[a]	Steady-State Pharmacokinetics of a Novel Extended-Release Metformin Formulation
Summary	The objective of this study was (1) to assess metformin steady-state pharmacokinetics when administered as extended-release tablet and (2) to compare it with those of metformin when administered as immediate-release tablet
Study type[a]	Bioavailability study
Number of participants[a]	16 (healthy volunteers)
Females (%)[a]	44
Age (years), Weight (kg), Height (cm)[a,b]	27 (19-40), 71 (53-103), 172 (160-193)
Inclusion Criteria[a]	• Adults<br>• Good health based upon recent medical history, laboratory determinations and physical examination<br>• Informed consent before enrolment
Exclusion criteria[a]	• Bodyweight >15% higher or lower than desirable weight-for-height range<br>• Blood donation within last 60 days<br>• History of clinically significant allergies to biguanides<br>• Exposure to any investigational agent within 60 days of enrolment or participation in any other clinical trials concurrent with this study<br>• Smoking more than 10 cigarettes per day<br>• History of gastrointestinal disease or recent use of medication that might affect the gastrointestinal tract<br>• Pregnant or nursing women
Study design[a]	Open label- randomised, multiple dose, five-regimen, two sequence clinical study (no wash-out between treatments)<br>1) Metformin extended-release 500 mg once daily (*n*=16)<br>2) Metformin extended-release 1000 mg once daily (*n*=16)<br>3) Metformin extended-release 1500 mg once daily (*n*=15)<br>4) Metformin extended-release 2000 mg once daily (*n*=8) or Metformin immediate-release 1000 mg twice daily (*n*=7)<br>5) Metformin extended-release 2000 mg one daily (*n*=7) or Metformin immediate-release 1000 mg twice daily (*n*=7)
Outcome measures[a]	C_{max} (immediate-release: evening dose), t_{max} (immediate-release: evening dose), $AUC_{0\text{-}infinity}$ on day 1, $AUC_{0\text{-}24}$ post-dose (for immediate-release: sum of $AUC_{0\text{-}12}$ morning and evening), $t_{1/2\beta}$ (immediate-release: evening dose)
Feeding state[a]	Fed
Metformin regimen[a]	Oral, tablet, once daily, 7 days (500, 1000, 1500, 2000 mg)<br>Oral, tablet, twice daily, 7 days (1000 mg)

ESM Table 1. *continued*

Study number	I
Maximum daily metformin dose[a]	2000 mg
Metformin formulation[a]	Extended-release (once daily administrations), immediate-release (twice daily administrations)
Daytime of metformin administration[a]	Once daily administrations:<br>• 19:30 hours<br>Twice daily administrations<br>• 19:30 hours on day 1, 07:30 hours and 19:30 hours on days 2-7
Metformin plasma sampling schedule	• Serial samples: Predose (19:30 hours), 1 h (20:30 hours), 2 h (21:30 hours), 4 h (23:30 hours), 6 h (01:30 hours), 8 h (03:30 hours), 12 h (07:30 hours), 16 h (11:30 hours), 20 h (15:30 hours), 24 h (19:30 hours) after drug administration (evening dose) on days 1, 6, 7, 13, 14, 20, 21, 27, 28, 34 and 35 (for various treatments)<br>• Immediate-release: Predose (07:30 hours), 1 h (08:30 hours), 2 h (09:30 hours), 4 h (11:30 hours), 6 h (13:30 hours), 8 h (15:30 hours), 12 h (19:30 hours), 13 h (20:30 hours), 14 h (21:30 hours), 16 h (23:30 hours), 18 h (01:30 hours), 20 h (03:30 hours) and 24 h (07:30 hours) after each morning dose on days 27, 28, 34 and 35<br>• Additional predose samples before evening dose on days 4, 5, 11, 12, 18, 19, 25, 26, 32 and 33

[a] Information extracted from the publication by Timmins et al. [1], [b] values for age, weight and height are given as mean (range). C_{max}, maximum plasma concentration; $t_{1/2\beta}$, terminal elimination half-life; t_{max}, time to reach C_{max}

ESM Table 2. Information on study II (metformin clinical phase I study by Boehringer Ingelheim [2])

**Study number**	**II**
ClinicalTrials.gov Identifier[a]	NCT02172248 (https://clinicaltrials.gov/ct2/show/NCT02172248)
Study ID[a]	1245.6
Official title[a]	Relative Bioavailability of Both BI 10773 and Metformin After Coadministration Compared to Multiple Oral Doses of BI 10773 (50 mg q.d.) Alone and Metformin (1000 mg b.i.d.) Alone to Healthy Male Volunteers (an Open-label, Randomised, Crossover, Clinical Phase I Study)
Study summary[a]	The objective was to investigate a possible drug-drug interaction between BI 10773 and metformin when co-administered as multiple oral doses. Therefore, the relative bioavailabilities of BI 10773 and metformin were determined when both drugs were given in combination compared with BI 10773 or metformin given alone.
Study type[a]	Interventional (Clinical Trial)
Number of participants[a]	16 (Healthy volunteers)
Females (%)[a]	0
Age (years), Weight (kg), Height (cm)[b,c]	32.2 (18-48), 81.3 (60-94), 180.1 (168-192)
GFR (ml/min/1.73m^2)[b]	82.7 (calculated from baseline serum creatinine according to MDRD equation)
Inclusion Criteria[a]	• Healthy male volunteers according to the following criteria: Based upon a complete medical history, including the physical examination, including the physical examination, vital signs (BP, PR), 12-lead ECG, clinical laboratory tests<br>• Age 18 to 50 years<br>• BMI 18.5 to 29.9 kg/m^2<br>• Signed and dated written informed consent prior to admission to the study in accordance with GCP and the local legislation
Exclusion criteria[a]	• Any finding of the medical examination (including BP, PR and ECG) deviating from normal and of clinical relevance<br>• Any evidence of a clinically relevant concomitant disease<br>• Gastrointestinal, hepatic, renal, respiratory, cardiovascular, metabolic, immunological or hormonal disorders<br>• Surgery of the gastrointestinal tract (except appendectomy)<br>• Diseases of the central nervous system (such as epilepsy) or psychiatric disorders or neurological disorders<br>• History of relevant orthostatic hypotension, fainting spells or blackouts<br>• Chronic or relevant acute infections<br>• History of relevant allergy/hypersensitivity (including allergy to drug or its excipients)<br>• Intake of drugs with a long half-life (> 24 hours) within at least one month or less than 10 half-lives of the respective drug prior to administration or during the trial<br>• Participation in another trial with an investigational drug within two months prior to administration or during the trial<br>• Smoker (> 10 cigarettes or > 3 cigars or > 3 pipes/day)<br>• Inability to refrain from smoking on trial days<br>• Alcohol abuse (more than 30 g/day)<br>• Drug abuse<br>• Blood donation (more than 100 ml within four weeks prior to administration or during the trial)<br>• Excessive physical activities (within one week prior to administration or during the trial)<br>• Any laboratory value outside the reference range that is of clinical relevance<br>• Inability to comply with dietary regimen of trial site

ESM Table 2. *continued*

**Study number**	**II**	
Study arms[a]	<u>Experimental: Sequence ABC</u><br>• Treatment A: BI 10773 once daily from day 1 to 5<br>• Treatment B: BI 10773 once daily from day 1 to 4 and metformin twice daily from day 1 to 3 and once in the morning on day 4<br>• Treatment C: metformin twice daily from day 1 to 3 and once in the morning on day 4	<u>Experimental: Sequence CAB</u><br>• Treatment C: metformin twice daily from day 1 to 3 and once in the morning on day 4<br>• Treatment A: BI 10773 once daily from day 1 to 5<br>• Treatment B: BI 10773 once daily from day 1 to 4 and metformin twice daily from day 1 to 3 and once in the morning on day 4
Outcome measures[a]	<u>Primary Outcome Measures</u><br>• $AUC_{\tau,ss}$, $C_{max,ss}$	<u>Secondary Outcome Measures</u><br>• $C_{24,N}$ of BI 10773, $C_{12,N}$ of metformin, $\lambda_{z,ss}$, $t_{½,ss}$, $t_{max,ss}$, $MRT_{po,ss}$, CL/F_{ss}, V_z/F_{ss}, $Ae_{t1\text{-}t2,ss}$, $fe_{t1\text{-}t2,ss}$, $CL_{R,ss}$ of BI 10773 and metformin, UGE<br>• Number of patients with abnormal findings in physical examination, number of patients with clinically significant changes in vital signs (BP, PR), number of patients with abnormal findings in 12-lead ECG, number of patients with abnormal changes in clinical laboratory tests, number of patients with adverse events, assessment of tolerability by investigator on a 4-point scale
Feeding state[b]	Fasted	
Metformin regimen[a]	Oral, 1000 mg, twice daily, 4 days	
Maximum daily metformin dose[a]	2000 mg	
Metformin formulation[b]	Immediate-release tablet	
Daytime of metformin administration[b,d]	08:00 hours, 20:00 hours (Days 1-3); 08:00 hours (Day 4)	
Metformin plasma sampling schedule[b,d]	<u>Days 2-3: C_{trough}</u><br>• 24 h (08:00 hours), 36 h (20:00 hours), 48 h (08:00 hours) and 60 h (20:00 hours) after first metformin administration	<u>Days 4-7: full profile</u><br>• 72 h (08:00 hours), 72.33 h (08:20 hours), 72.67 h (08:40 hours), 73 h (09:00 hours), 74 h (10:00 hours), 74.5 h (10:30 hours), 75 h (11:00 hours), 76 h (12:00 hours), 78 h (14:00 hours), 80 h (16:00 hours), 82 h (18:00 hours), 84 h (20:00 hours), 86 h (22:00 hours), 96 h (08:00 hours), 108 h (20:00 hours), 120 h (08:00 hours) and 144 h (08:00 hours) after first metformin administration

[a] Information extracted from https://clinicaltrials.gov/ct2/show/NCT02172248, [b] information extracted from the study report, [c] values for age, weight and height are given as mean (range), [d] planned time, actual administration and sampling time is known and was used for model development. $Ae_{t1\text{-}t2,ss}$, amount of analyte eliminated in urine at steady state over a uniform dosing interval τ; $AUC_{\tau,s}$, area under the concentration-time curve of the analyte in plasma at steady state over a uniform dosing interval τ; b.i.d., twice daily; BP, blood pressure; $C_{12,N}$, concentration of analyte in plasma at 12 hours post-drug administration after administration of the Nth dose; $C_{24,N}$, concentration of analyte in plasma at 24 hours post-drug administration after administration of the Nth dose; CL/F_{ss}, apparent clearance of the analyte in the plasma after extravascular administration at steady state; $CL_{R,ss}$, renal clearance of the analyte at steady state; $C_{max,ss}$, maximum measured concentration of the analyte in plasma at steady state over a uniform dosing interval τ; C_{trough}, trough plasma concentration; ECG, electrocardiogram; $fe_{t1\text{-}t2,ss}$, fraction of analyte excreted unchanged in urine at steady state over a uniform dosing interval τ; GCP, good clinical practice; $MRT_{po,ss}$, mean residence time of the analyte in the body at steady state after oral administration; PR, pulse rate; q.d., once daily; $t_{½,ss}$, terminal half-life of the analyte in plasma at steady state; $t_{max,ss}$, time from last dosing to maximum concentration of the analyte in plasma at steady state over a uniform dosing interval τ; UGE, urinary glucose excretion; V_z/F_{ss}, apparent volume of distribution during the terminal phase λ_z at steady state following extravascular administration; $\lambda_{z,ss}$, terminal half-life of the analyte in plasma

ESM Table 3. Information on study III (metformin clinical phase I study by Boehringer Ingelheim [3])

**Study number**	**III**
ClinicalTrials.gov Identifier[a]	NCT02183506 (https://clinicaltrials.gov/ct2/show/NCT02183506)
Study ID[a]	1218.4
Official title[a]	Bioavailability of Both BI 1356 BS and Metformin After Co-administration Compared to the Bioavailability of Multiple Oral Doses of BI 1356 BS 10 mg Daily Alone and Metformin 850 mg Three Times a Day Alone in Healthy Male Volunteers (an Open-label, Randomized, Crossover Study)
Study summary[a]	Investigate the bioavailability of BI 1356 BS and of metformin after concomitant multiple oral administration of 10 mg BI 1356 BS tablets and 3 x 850 mg metformin in comparison to BI 1356 BS and metformin given alone
Study type[a]	Interventional (Clinical Trial)
Number of participants[a]	16 (Healthy volunteers)
Females (%)[a]	0
Age (years), Weight (kg), Height (cm)[b,c]	32.2 (22-44), 81.8 (62-106), 176.6 (163-190)
GFR (ml/min/1.73m^2)[b]	86.2 (calculated from baseline serum creatinine according to MDRD equation)
Inclusion Criteria[a]	• Healthy males according to the following criteria, based upon a complete medical history, including the physical examination, vital signs (BP, PR), 12-lead ECG, clinical laboratory tests, no finding deviating from normal and of clinical relevance, no evidence of a clinically relevant concomitant disease<br>• Age ≥ 21 and Age ≤ 50 years<br>• BMI (Body Mass Index) ≥ 18.5 and ≤ 29.9 kg/m^2<br>• Ability to give signed and dated written informed consent prior to admission to the study in accordance with GCP and the local legislation
Exclusion criteria[a]	• Gastrointestinal, hepatic, renal, respiratory, cardiovascular, metabolic, immunological or hormonal disorders<br>• Surgery of the gastrointestinal tract (except appendectomy)<br>• Diseases of the central nervous system (such as epilepsy) or psychiatric disorders or neurological disorders<br>• History of relevant orthostatic hypotension, fainting spells or blackouts<br>• Chronic or relevant acute infections<br>• History of allergy/hypersensitivity (including drug allergy) which is deemed relevant to the trial by the investigator<br>• Intake of drugs with a long half-life (>24 hours) within one month or less than 10 half-lives of the respective drug prior to administration or during the conduct of this trial (review with clinical monitor if there is a question)<br>• Use of drugs which might reasonably influence the results of the trial (based on knowledge at the time of protocol preparation) within 10 days prior to administration or during the conduct of this trial<br>• Participation in another trial with an investigational drug within two months prior to administration or during the conduct of this trial<br>• Smoker (more than 10 cigarettes or 3 cigars or 3 pipes per day)<br>• Inability to refrain from smoking during the conduct of this trial<br>• Alcohol abuse (more than 60 g/day)<br>• Drug abuse<br>• Blood donation (more than 100 ml within four weeks prior to administration or during the conduct of this trial)<br>• Excessive physical activities (within one week prior to administration or during the conduct of this trial)<br>• Any laboratory value outside the normal reference range that is of clinical relevance<br>• Inability to comply with the dietary regimen of the study center<br>• No adequate contraception (condom use plus another form of contraception e.g. spermicide, oral contraceptive taken by female partner, sterilisation) during the whole study period from the time of the first intake of study drug until one month after the last intake of drug

ESM Table 3. *continued*

Study number	III	
Study arms[a]	Daily administration of BI 1356 BS alone (day 1 to day 6) followed by the combined treatment of BI 1356 BS with metformin (day 7 to day 9)	
Outcome measures[a]	Primary Outcome Measures<br>• AUC of the analytes in plasma at different time points, C_{max} of the analytes in plasma at different time points	Secondary Outcome Measures<br>• $t_{max,ss}$, $C_{min,ss}$, $\lambda_{z,ss}$, $t_{1/2,ss}$, $MRT_{po,ss}$, CL/F_{ss}, V_z/F_{ss}, DPP-IV activity, $fe_{\tau,ss}$, $CL_{R,ss}$<br>• Number of patients with adverse events, number of patients with clinically abnormal changes in laboratory values, number of patients with clinically relevant changes in vital signs
Feeding state[b]	Fed	
Metformin regimen[a]	Oral, 850 mg, three times daily, 3 days	
Maximum daily metformin dose[a]	2550 mg	
Metformin formulation[b]	Immediate-release tablet	
Daytime of metformin administration[b,d]	07:30 hours, 15:30 hours, 23:30 hours (Days 1-2); 07:30 hours (Day 3)	
Metformin plasma sampling schedule[b,d]	Day 2: C_{trough}<br>• 31.83 h (15:20 hours) and 39.83 h (23:20 hours) after first metformin administration	Days 3-5: full profile<br>• 47.83 h (07:20 hours), 48.25 h (07:45 hours), 48.5 h (08:00 hours), 48.75 h (08:15 hours), 49 h (08:30 hours), 49.5 h (09:00 hours), 50 h (09:30 hours), 50.5 h (10:00 hours), 51 h (10:30 hours), 52 h (11:30 hours), 54 h (13:30 hours), 56 h (15:30 hours), 60 h (19:30 hours), 72 h (07:30 hours) and 96 h (07:30 hours) after first metformin administration

[a] Information extracted from https://clinicaltrials.gov/ct2/show/NCT02183506, [b] information extracted from the study report, [c] values for age, weight and height are given as mean (range), [d] planned time, actual administration and sampling time is known and was used for model development. BP, blood pressure; CL/F_{ss}, apparent clearance of the analyte in the plasma after extravascular administration at steady state; $CL_{R,ss}$, renal clearance of the analyte at steady state; $C_{min,ss}$, minimum concentration of the analytes in plasma at steady state; C_{max}, maximum concentration; C_{trough}, trough plasma concentration; DPP-IV, dipeptidylpeptidase 4; ECG, electrocardiogram; $fe_{\tau,ss}$, fraction of the dose excreted unchanged in urine at steady state; $fe_{t1-t2,ss}$, fraction of analyte excreted unchanged in urine at steady state over a uniform dosing interval τ; GCP, good clinical practice; $MRT_{po,ss}$, mean residence time of the analyte in the body at steady state after oral administration; PR, pulse rate; $t_{½,ss}$, terminal half-life of the analyte in plasma at steady state; $t_{max,ss}$, time from last dosing to maximum concentration of the analyte in plasma at steady state over a uniform dosing interval τ; UGE, urinary glucose excretion; V_z/F_{ss}, apparent volume of distribution during the terminal phase λ_z at steady state following extravascular administration; $\lambda_{z,ss}$, terminal half-life of the analyte in plasma

ESM Table 4. Information on study IV (metformin clinical phase I study by Boehringer Ingelheim [4])

Study number	IV
ClinicalTrials.gov Identifier[a]	NCT01845077 (https://clinicaltrials.gov/ct2/show/NCT01845077)
Study ID[a]	1288.8
Official title[a]	Relative Bioavailability of Two Newly Developed Extended Release FDC Tablet Strengths (5mg/1000mg and 2.5 mg/750 mg) of Linagliptin/Metformin Extended Release Compared With the Free Combination of Linagliptin and Metformin Extended Release in Healthy Subjects (an Open-label, Randomised, Single Dose, Two-way Crossover Study)
Study summary[a]	The purpose of the trial is to demonstrate the relative bioavailability of 2 newly developed fixed dose combination (FDC) tablets containing linagliptin & metformin and the single tablets of linagliptin and metformin when administered singularly
Study type[a]	Interventional (Clinical Trial)
Number of participants[a]	72 (Healthy volunteers)
Females (%)[a]	42
Age (years), Weight (kg), Height (cm)[b,c]	31.3 (18-49), 72.8 (47-100), 169.5 (147-198)
Inclusion Criteria[a]	• Healthy males or females<br>• Age 18 -50 years<br>• BMI 18.5 to 29.9 kg/m^2<br>• Subjects must be able to understand and comply with study requirements
Exclusion criteria[a]	Any deviation from healthy condition
Study arms[a]	FDC1000 Fasted vs. L+M1000 Fasted<br>• 1 FDC tablet (5 mg linagliptin/1000mg metformin FDC) vs. 3 single tablets (1 x 5 mg linagliptin + 2 x 500 mg metformin) (and vice versa)<br>FDC1000 Fed vs. L+M1000 Fed<br>• 1 FDC tablet (5 mg linagliptin/1000mg metformin FDC) vs. 3 single tablets (1 x 5 mg linagliptin + 2 x 500 mg metformin) (and vice versa)<br>FDC1500 Fasted vs. L+M1500 Fasted<br>• 2 FDC tablets (2 x 2.5 mg linagliptin/750mg metformin FDC) vs. 4 single tablets (1 x 5 mg linagliptin + 3 x 500 mg metformin) (and vice versa)
Outcome measures[a]	Primary Outcome Measures<br>• $AUC_{0\text{-}72}$ and C_{max} of linagliptin , $AUC_{0\text{-}tz}$ and C_{max} of metformin<br>Secondary Outcome Measures<br>• $AUC_{0\text{-}infinity}$ of linagliptin, $AUC_{0\text{-}infinity}$ of metformin
Feeding state (Metformin dose)[a]	Fasted (1000mg), Fed (1000mg), Fasted (1500mg)
Metformin dosing regimen[a]	Oral, single dose
Maximum daily metformin dose[a]	1000/1500 mg
Metformin formulation[a]	Extended-release tablet
Daytime of metformin administration[b,d]	08:00 hours
Metformin plasma sampling schedule[a,b,d]	20 min (08:20 hours), 40 min (08:40 hours), 1 h (09:00 hours), 1.5 h (09:30 hours), 2 h (10:00 hours), 3 h (11:00 hours), 4 h (12:00 hours), 5 h (13:00 hours), 6 h (14:00 hours), 8 h (16:00 hours), 10 h (18:00 hours), 12 h (20:00 hours), 16 h (00:00 hours), 24 h (08:00 hours), 36 h (20:00 hours), 48 h (08:00 hours) and 72 h (08:00 hours) after metformin administration

[a] Information extracted from https://clinicaltrials.gov/ct2/show/NCT01845077, [b] information extracted from the study report, [c] values for age, weight and height are given as mean (range), [d] planned time, actual administration and sampling time is known and was used for model development. $AUC_{0\text{-}72}$, area under the concentration-time curve in plasma over the time interval 0 to 72 hours; $AUC_{0\text{-}infinity}$, area under the concentration-time curve in plasma over the time interval from 0 extrapolated to infinity based on predicted last concentration values; $AUC_{0\text{-}tz}$, area under the concentration-time curve in plasma over the time interval from 0 to the last quantifiable data point; C_{max}, maximum concentration; FDC, fixed dose combination

ESM Table 5. Information on study V (metformin clinical phase I study by Boehringer Ingelheim [5])

**Study number**	**V**
ClinicalTrials.gov Identifier[a]	NCT01975220 (https://clinicaltrials.gov/ct2/show/NCT01975220)
Study ID[a]	1276.13
Official title[a]	Relative Bioavailability of Two Newly Developed FDC Tablet Strengths (25mg/1000mg and 12.5mg/750mg) of Empagliflozin/Metformin Extended Release Compared With the Free Combination of Empagliflozin and Metformin Extended Release in Healthy Subjects (an Open-label, Randomised, Single Dose, Two-way Crossover Study)
Study summary[a]	The purpose of this trial is to demonstrate the relative bioavailability of 2 newly developed (FDC) tablets containing empagliflozin & metformin and the single tablets of empagliflozin and metformin when administered singularly
Study type[a]	Interventional (Clinical Trial)
Number of participants[a]	72 (Healthy volunteers)
Females (%)[a]	42
Age (years), Weight (kg), Height (cm)[b,c]	32.5 (19-50), 74.3 (50-115), 169.5 (149-198)
Inclusion Criteria[a]	• Healthy males or females<br>• Age 18-50 years<br>• BMI 18.5 to 29.9 kg/m^2<br>• Subjects must be able to understand and comply with study requirements
Exclusion criteria[a]	Any deviation from healthy condition
Study arms[a]	High Dose, Fasted<br>• 1 FDC tablet (25 mg empagliflozin/1000 mg metformin FDC) vs. 3 single tablets (25 mg empagliflozin + 2 x 500 mg metformin) (and vice versa)<br>High Dose, Fed<br>• 1 FDC tablet (25 mg empagliflozin/1000 mg metformin) vs. 3 single tablets (1 x 25 mg empagliflozin + 2 x 500 mg metformin) (and vice versa)<br>Low Dose, Fasted<br>• 2 FDC tablets (2 x 12.5 mg empagliflozin/750 mg metformin) vs. 4 single tablets (1 x 25 mg empagliflozin + 3 x 500 mg metformin) (and vice versa)
Outcome measures[a]	Primary Outcome Measures<br>• $AUC_{0\text{-}tz}$ and C_{max} of empagliflozin, $AUC_{0\text{-}tz}$ and C_{max} of metformin<br>Secondary Outcome Measures<br>• $AUC_{0\text{-}infinity}$ of empagliflozin, $AUC_{0\text{-}infinity}$ of metformin
Feeding state (Metformin dose)[a]	Fasted (1000mg), Fed (1000mg), Fasted (1500mg)
Metformin dosing regimen[a]	Oral, single dose
Maximum daily metformin dose[a]	1000/1500 mg
Metformin formulation[a]	Extended-release tablet
Daytime of metformin administration[b,d]	08:00 hours
Metformin plasma sampling schedule[a,b,d]	20 min (08:20 hours), 40 min (08:40 hours), 1 h (09:00 hours), 1.33 h (09:20 hours), 1.67 h (09:40 hours), 2 h (10:00 hours), 2.5 h (10:30 hours), 3 h (11:00 hours), 4 h (12:00 hours), 5 h (13:00 hours), 6 h (14:00 hours), 7 h (15:00 hours), 8 h (16:00 hours), 9 h (17:00 hours), 10 h (18:00 hours), 12 h (20:00 hours), 16 h (00:00 hours), 24 h (08:00 hours), 36 h (20:00 hours), 48 h (08:00 hours) and 72 h (08:00 hours) after metformin administration

[a] Information extracted from https://clinicaltrials.gov/ct2/show/NCT01975220, [b] Information extracted from the study report, [c] values for age, weight and height are given as mean (range), [d] planned time, actual administration and sampling time is known and was used for model development. $AUC_{0\text{-}72}$, area under the concentration-time curve in plasma over the time interval 0 to 72 hours; $AUC_{0\text{-}infinity}$, area under the concentration-time curve in plasma over the time interval from 0 extrapolated to infinity based on predicted last concentration values; $AUC_{0\text{-}tz}$, area under the concentration-time curve in plasma over the time interval from 0 to the last quantifiable data point; C_{max}, maximum concentration; FDC, fixed dose combination

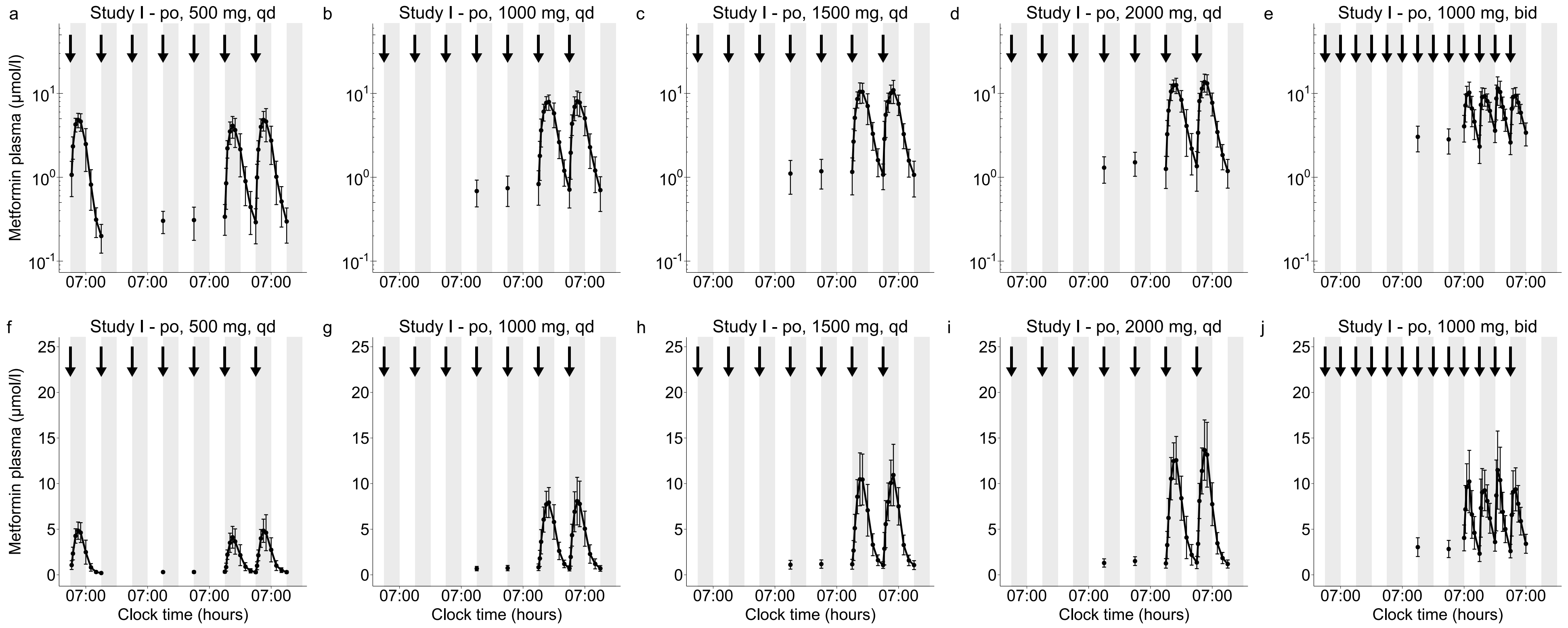

ESM Fig 1. Mean observed plasma concentration-time profiles for all administered regimen of study I [1]. Upper panel: semilogarithmic plots (concentration presented on decadic logarithm scale), lower panel: linear plots. Metformin was administered as (**a**—**d**) and (**f**—**i**) extended-release or (**e**, **j**) immediate-release tablet. Data are shown as arithmetic means ± SD. Black arrows indicate drug administration. Grey areas indicate night-time. bid, twice daily; po, oral; qd, once daily

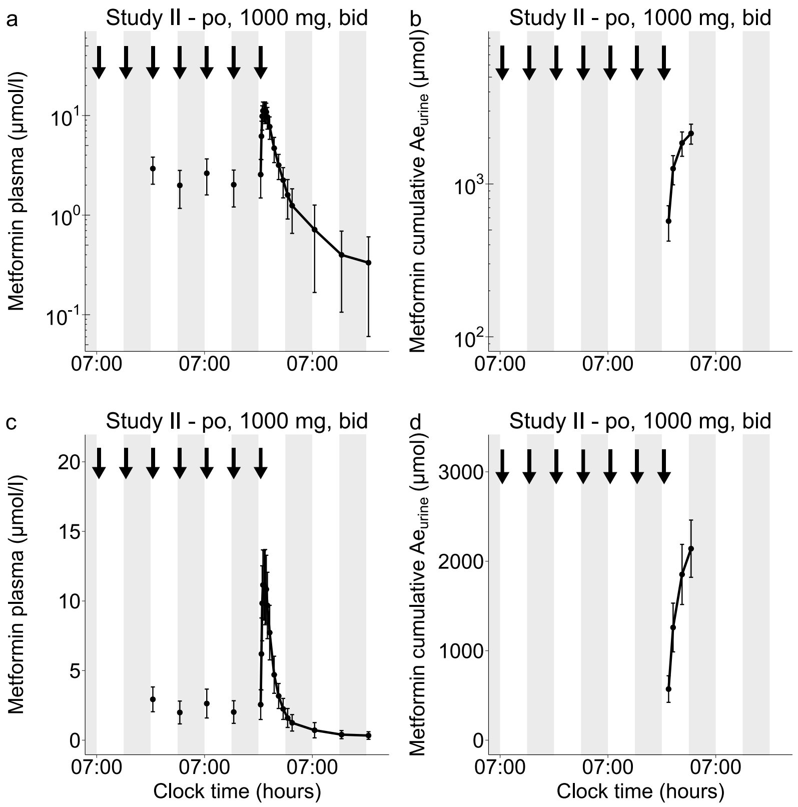

ESM Fig 2. Mean observed (**a**, **c**) plasma and (**b**, **d**) urine concentration-time profiles of study II [2]. Upper panel: semilogarithmic plots (concentration presented on decadic logarithm scale), lower panel: linear plots. Metformin was administered as immediate-release tablet. Data are shown as arithmetic means ± SD. Black arrows indicate drug administration. Grey areas indicate night-time. Ae_{urine}, amount excreted unchanged in urine; bid, twice daily; po, oral

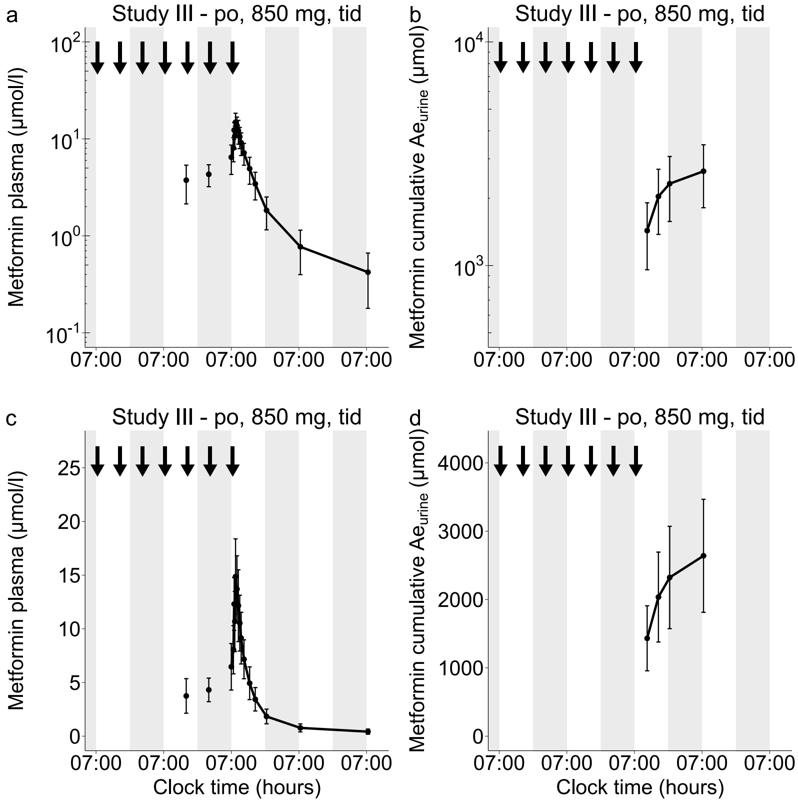

ESM Fig 3. Mean observed (**a**, **c**) plasma and (**b**, **d**) urine concentration-time profiles of study III [3]. Upper panel: semilogarithmic plots (concentration presented on decadic logarithm scale), lower panel: linear plots. Metformin was administered as immediate-release tablet. Data are shown as arithmetic means ± SD. Black arrows indicate drug administration. Grey areas indicate night-time. Ae_{urine}, amount excreted unchanged in urine; po, oral; tid, three times daily

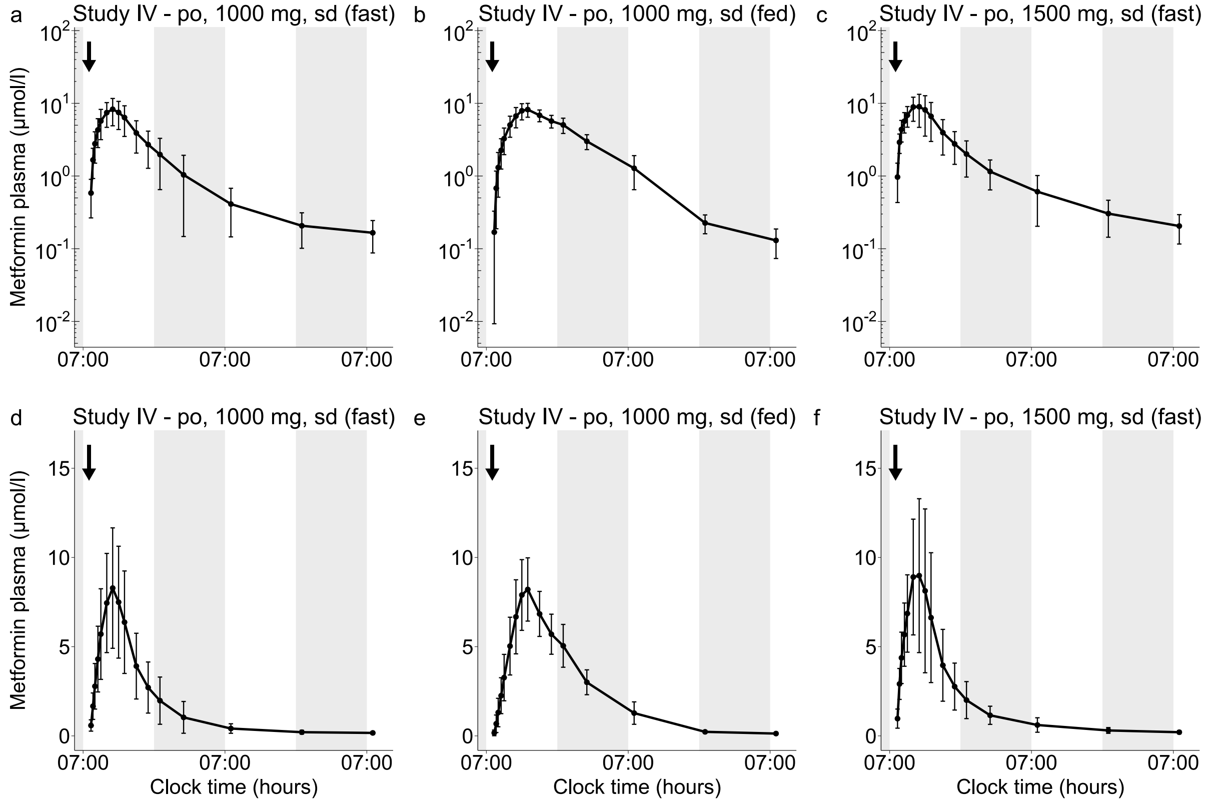

ESM Fig 4. Mean observed plasma concentration-time profiles of study IV [4]. Upper panel: semilogarithmic plots (concentration presented on decadic logarithm scale), lower panel: linear plots. Metformin was administered as extended-release tablet. Data are shown as arithmetic means ± SD. Black arrows indicate drug administration. Grey areas indicate night-time. fast, fasted state; fed, fed state; po, oral; sd, single dose

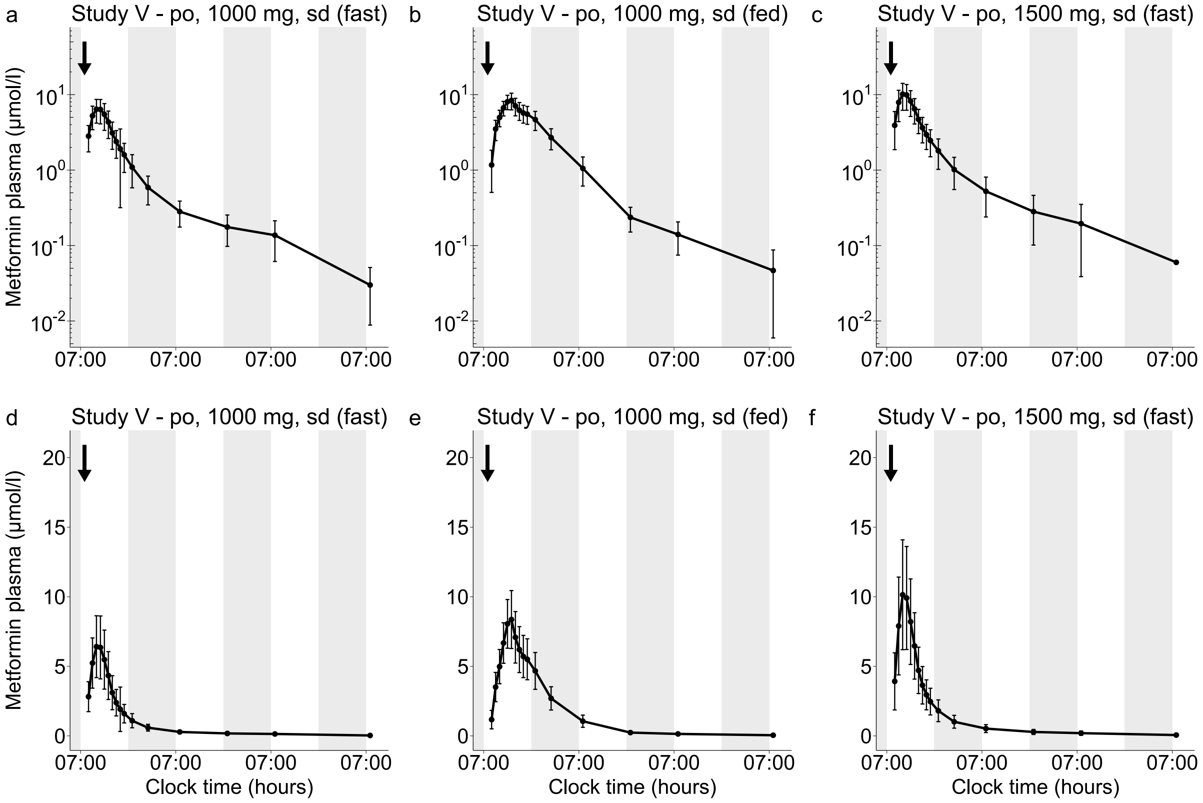

ESM Fig 5. Mean observed plasma concentration-time profiles of study V [5]. Upper panel: semilogarithmic plots (concentration presented on decadic logarithm scale), lower panel: linear plots. Metformin was administered as extended-release tablet. Data are shown as arithmetic means ± SD. Black arrows indicate drug administration. Grey areas indicate night-time. fast, fasted state; fed, fed state; po, oral; sd, single dose

1.2 Statistical analysis

Individual plasma measurements were analysed separately for differences between trough plasma concentration (C_{trough}) values measured immediately before the next dose in the morning (‘$C_{trough,morning}$’) and the evening (‘$C_{trough,evening}$’). $C_{trough,morning}$ and $C_{trough,evening}$ were calculated average for each individual, and differences were compared using a paired *t* test. Additionally, a linear mixed model with random effect on study participant was applied to account for intra- and interindividual variability, including unequal numbers of measurements in the morning and the evening for each individual. The same procedure was applied to compare maximum plasma concentration (C_{max}) values measured after the morning dose (‘$C_{max,morning}$’) and the evening dose (‘$C_{max,evening}$’). For all statistical analyses, the significance level α was set to 0.05 (5%).

1.3 Non-linear mixed effects (NLME) pharmacokinetic modelling

NLME pharmacokinetic modelling was performed using NONMEM (NONMEM version 7.4.3, ICON Development Solutions, Ellicott City, MD, USA), allowing estimation of population medians for pharmacokinetic model parameters with simultaneous quantification of interindividual variability and residual (unexplained) variability. Calculation of pharmacokinetic parameters, quantitative model performance analysis, and generation of plots were accomplished using R 4.0.2 (R Core Team. R: A language and environment for statistical computing. R Foundation for Statistical Computing, Vienna, Austria) and RStudio 1.2.5033 (RStudio, Inc., Boston, MA, USA).

Model selection and hypothesis rating (model with time-of-day variation) was based on the precision of parameter estimates, the objective function value (OFV) provided by NONMEM [6] and visual inspection of goodness-of-fit plots (plotting predicted plasma and urine concentrations vs. observed values as well as conditional weighted residuals (CWRES) vs. time or predicted concentrations). One nested model was considered superior to another when the OFV was reduced by 3.84 units (Chi^2, $p <$ 0.05, 1 degree of freedom) [7]. The First-Order Conditional Estimation with Interaction (FOCE-I) method was applied. To additionally evaluate the model performance of the model without versus with time-of-day variation, predicted and observed C_{trough} and C_{max} ratios (morning vs. evening) were compared including calculation of geometric mean fold errors (GMFEs) of C_{trough} and C_{max} ratio predictions as quantitative measure according to ESM Equation 1.

$$\text{GMFE} = 10^{x}; \qquad x = \frac{1}{m}\sum_{i=1}^{m}\left|\log_{10}\left(\frac{\text{predicted PK parameter}_i}{\text{observed PK parameter}_i}\right)\right| \qquad (1)$$

where predicted PK parameter$_i$ = predicted C_{trough} or C_{max} ratio, observed PK parameter$_i$ = corresponding observed C_{trough} or C_{max} ratio and m = number of studies. Overall GMFEs of ≤ 2 were considered reasonable predictions.

1.4 Literature-informed mechanistic physiologically based pharmacokinetic (PBPK) modelling

The previously published PBPK model [8] was developed in PK-Sim® and during this analysis extended in MoBi® (Open systems pharmacology suite 8.0, http://www.open-systems-pharmacology.org/). Calculation of pharmacokinetic parameters, quantitative model performance analysis, and generation of plots were accomplished using R 4.0.2 (R Core Team. R: A language and environment for statistical computing. R Foundation for Statistical Computing, Vienna, Austria) and RStudio 1.2.5033 (RStudio, Inc., Boston, MA, USA).

Virtual twins of study individuals were generated according to the demographic information, with corresponding ethnicity, sex, age, body weight, height and GFR, if reported. Metformin transporters were implemented in agreement with current literature, utilising the PK-Sim® expression database [9] to define their relative expression in the different organs of the body. Details on the expression of drug transporters implemented to model the pharmacokinetics of metformin are summarised in ESM Table 6. In all virtual individuals, enterohepatic circulation (EHC) was enabled (EHC continuous fraction set to 1) by assuming a continuous flow of the bile to the duodenum.

Model performance was evaluated by (1) comparison of predicted and observed plasma concentration time-profiles, (2) comparison of predicted and observed plasma concentration values in goodness-of-fit plots, (3) calculation of mean relative deviations (MRDs) of plasma concentration predictions according to ESM Equation 2, (4) comparison of predicted and observed C_{trough} and C_{max} ratios (morning vs. evening) and (5) calculation of GMFEs of C_{trough} and C_{max} ratio predictions.

$$\mathrm{MRD} = 10^{x}; \qquad x = \sqrt{\frac{1}{k}\sum_{i=1}^{k}\left(\log_{10} c_{predicted,i} - \log_{10} c_{observed,i}\right)^{2}} \qquad (2)$$

where $c_{predicted,i}$ = predicted plasma concentration, $c_{observed,i}$ = corresponding observed plasma concentration and k = number of observed values. Overall MRD values ≤ 2 were considered reasonable predictions.

ESM Table 6. System-dependent parameters

Transporter	Ref. Conc. (µmol/l)[a]	Expression profile[b]	Localisation	Direction	Half life [h]
MATE1	0.13[c] [10, 11]	Kidney only [12, 13]	Apical	Efflux	36 (liver)
OCT1	0.16[d] [14, 15]	Array [16][e]	Basolateral[f]	Influx	36 (liver), 23 (intestine)
OCT2	0.19[c] [10, 11]	EST [17]	Basolateral	Influx	36 (liver)
PMAT	1.00[g] [18]	RT-PCR [19][e]	Basolateral[f]	Influx	36 (liver), 23 (intestine)

Array, ArrayExpress measured expression profile; EST, expressed sequence tag measured expression profile; MATE, multidrug and toxin extrusion protein; OCT, organic cation transporter; PMAT, plasma membrane monoamine transporter; ref. conc., reference concentration; RT-PCR, reverse transcription-polymerase chain reaction measured expression profile

[a] mean reference concentration µmol/l in the tissue of highest expression

[b] relative expression in the different organs (PK-Sim expression database profile)

[c] calculated from transporter per mg membrane protein x 26.2 mg human kidney microsomal protein per g kidney [10]

[d] calculated from transporter per mg membrane protein x 37.0 mg membrane protein per g liver [14]

[e] large intestinal mucosa → 0

[f] apical in enterocytes

[g] transport rate constant (k_{cat}) was optimised according to [18]

2 ESM Results

2.1 Statistical analysis

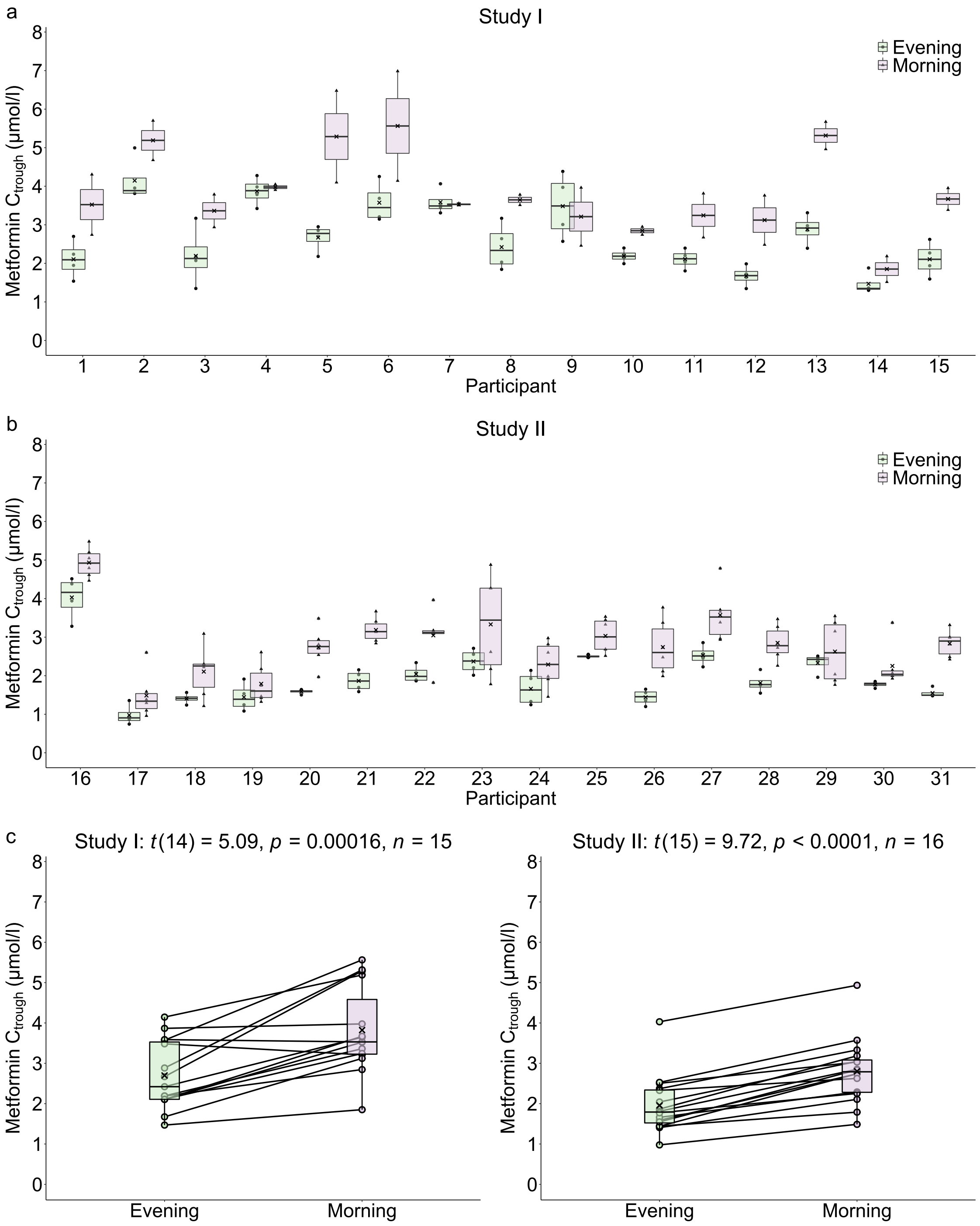

Fig 6. Intra- and interindividual variability of trough plasma concentration (C_{trough}) measurements from studies I and II [1, 2], including paired *t* test results. Boxes represent the distance between first and third quartiles (IQR). Whiskers range from smallest to highest value (< 1.5 × IQR).

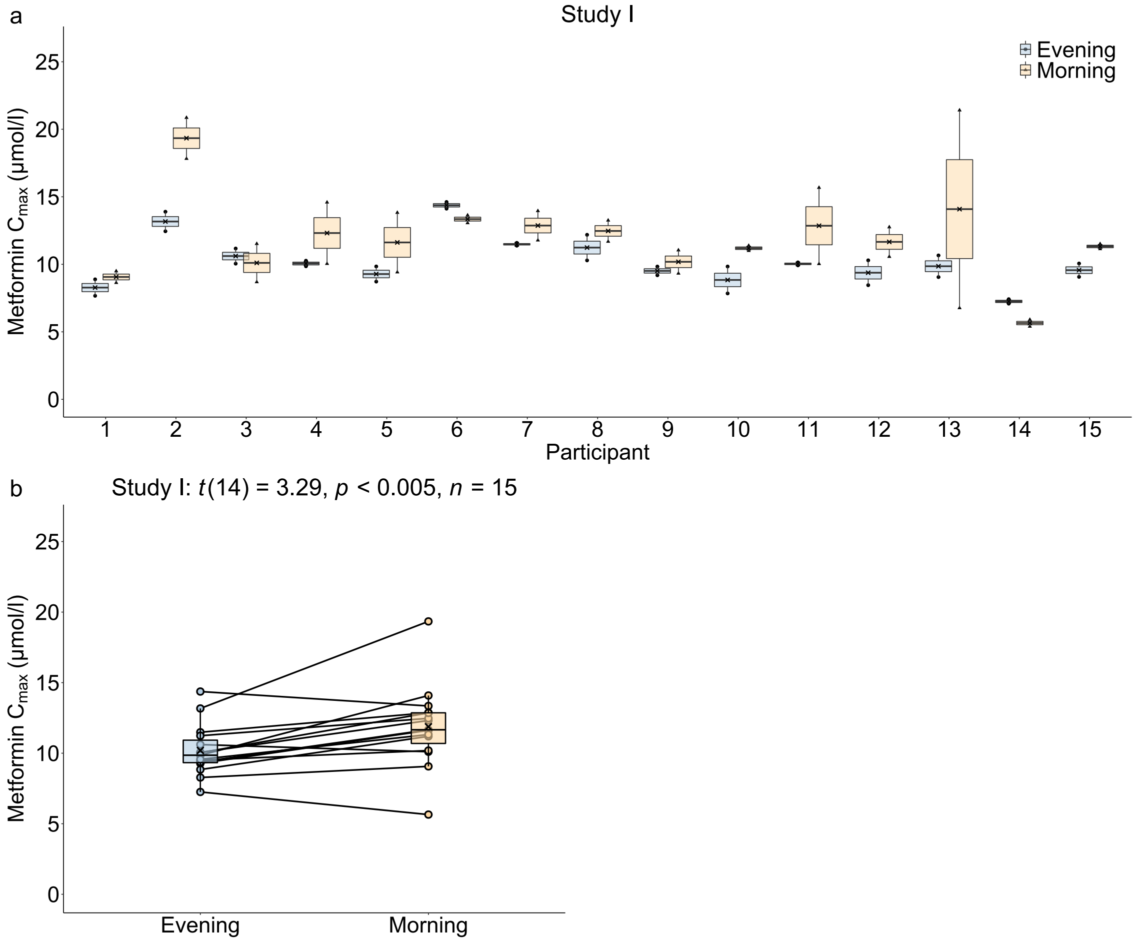

Fig 7. Intra- and interindividual variability of maximum plasma concentration (C_{max}) measurements from study I [1], including paired *t* test results. Boxes represent the distance between first and third quartiles (IQR). Whiskers range from smallest to highest value (< 1.5 × IQR).

2.2 NLME pharmacokinetic modelling

The volumes of distribution were 41.1 l for central (V_2) and 129 l for peripheral (V_3) compartments, respectively. The physiologically described rhythm of the GFR was implemented by multiplication of a sine function with clearance. The amplitude and a time shift of the sine function were estimated as 21% and 12.3 h (acrophase at 17:43 hours), respectively. Interindividual variability was found on the clearance, the volume of distribution and the bioavailability. Implementation of food, formulation and dose as significant covariates partly explained the interindividual variability and the related parameters were reduced about 14.2%, 75.0% and 51.5% for clearance, volume of distribution and bioavailability, respectively. Administration after food intake leads to a 1.91-fold higher bioavailability and a 0.63-fold lower absorption rate constant but 5.10-fold increased release duration for the extended-release formulation. The bioavailability of the extended-release formulation was 1.09-fold higher compared to the immediate-release formulation. The dose was implemented as a covariate using an exponential function according to ESM Equation 3, leading to a decreased bioavailability by administration of higher doses metformin. All estimated parameter values are summarised in ESM Table 7. All model parameters were precisely estimated with residual standard errors < 25%. Observed versus model predicted metformin concentrations are randomly distributed around the line of identity, indicating good descriptive properties of the final pharmacokinetic model.

$$F1 = \text{estimated rel. } F1 \times \left(\frac{\text{dose}}{1000}\right)^{-0.117} \quad (3)$$

where F1 = absolute bioavailability, estimated rel. F1 = estimated relative bioavailability and dose = metformin dose.

ESM Table 7. NLME pharmacokinetic model parameters

Parameter	Unit	Value (RSE)		Description
		**Without time-of-day variation**	**With time-of-day variation**	
ΔOBV			-660.39	Drop in objective function value
Fixed effects				
CL	l/h	27.7 (14%)	28.9 (10%)	Clearance
V_2	l	19.1 (15%)	41.1 (9%)	Central volume of distribution
k_a	1/h	0.261 (3%)	0.280 (4%)	Absorption rate constant
V_3	l	133 (15%)	129 (12%)	Peripheral volume of distribution
Q	l/h	6.18 (16%)	6.01 (11%)	Intercompartimental clearance
F1	%	0.181 (19%)	0.191 (19%)	Absolute bioavailability
TDEL	h	-	12.3 (1%)	Timeshift of the sine function
AMP	-	-	0.21 (7%)	Amplitude of the sine function
D1_bioequivalence	h	2.49 (9%)	5.12 (3%)	Duration of the release of the extended-release formulation (study I)
D1_phase_I	h	2.38 (7%)	1.18 (6%)	Duration of the release of the extended-release formulation (studies IV and V)
Factor_D1_fed	-	2.54 (8.3%)	5.12 (3%)	
Factor_F1_ER	-	1 (3%)	1.09 (3%)	
Factor_k_a_fed	-	0.681 (4%)	0.631 (5%)	
Exponent_F1_dose	-	-0.134 (16%)	-0.117 (17%)	
Random effects				
IIV CL	%CV	68.5 (7%)	62.5 (8%)	Interindividual variability on clearance
IIV V_2	%CV	137.3 (7%)	91 (11%)	Interindividual variability on volume of distribution
IIV F1	%CV	61 (9%)	63.3 (15%)	Interindividual variability on bioavailabitiy
Residual effects				
PRV	%	32 (4%)	30.1 (9%)	Proportional residual variability of bioequivalence study (plasma concentration) (study I)
PRV	%	32.9 (2%)	31.6 (2 %)	Proportional residual variability of phase I trials (plasma concentration) (studies II–V)
PRV	%	56.8 (12%)	55 (11%)	Proportional residual variability of phase I trials (urine concentration) (studies II and III)

RSE, relative standard error

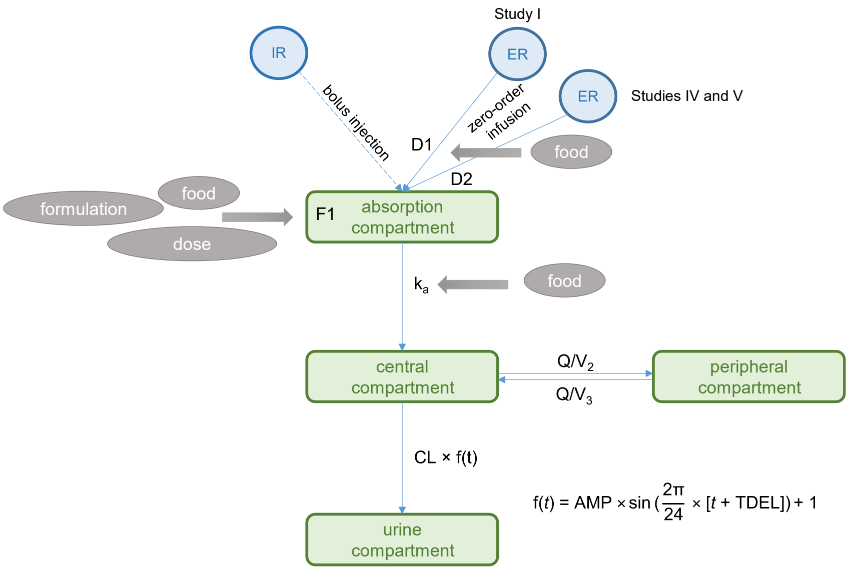

Fig 8. Structure of the final metformin NLME pharmacokinetic model. AMP, amplitude; CL, clearance; D, duration of release of the formulation; ER, extended-release; F1, absolute bioavailability; IR, immediate-release; k_a, absorption rate constant; Q, intercompartimental clearance; t, time; TDEL, shift in time; V, volume of distribution

2.2.1 NLME pharmacokinetic model plots

2.2.1.1 Metformin plasma concentration-time profiles

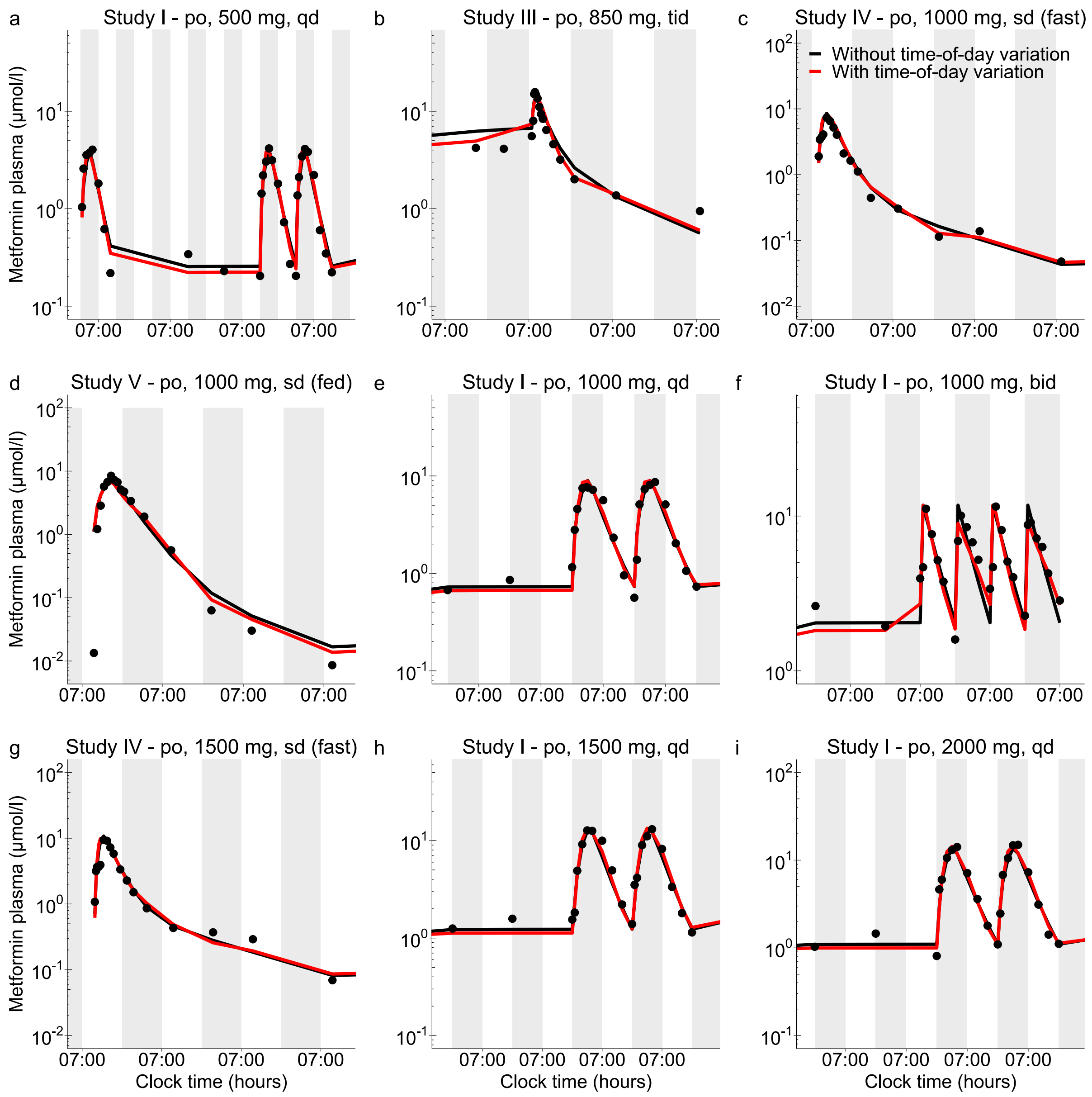

ESM Fig 9. Exemplary individual plasma concentration-time profiles for each administered regimen (semilogarithmic plots, i.e. concentration presented on decadic logarithm scale). Metformin was administered as (**a**, **c**, **d**, **e**, **g**, **h**, **i**) extended-release or (**b**, **f**) immediate-release tablet. Observed data from studies I-V [1–5] are shown as dots, predictions are shown as lines (black: without time-of-day variation, red: with time-of-day variation). Grey areas indicate night-time. bid, twice daily; fast, fasted state; fed, fed state; po, oral; sd, single dose; tid, three times daily; qd, once daily

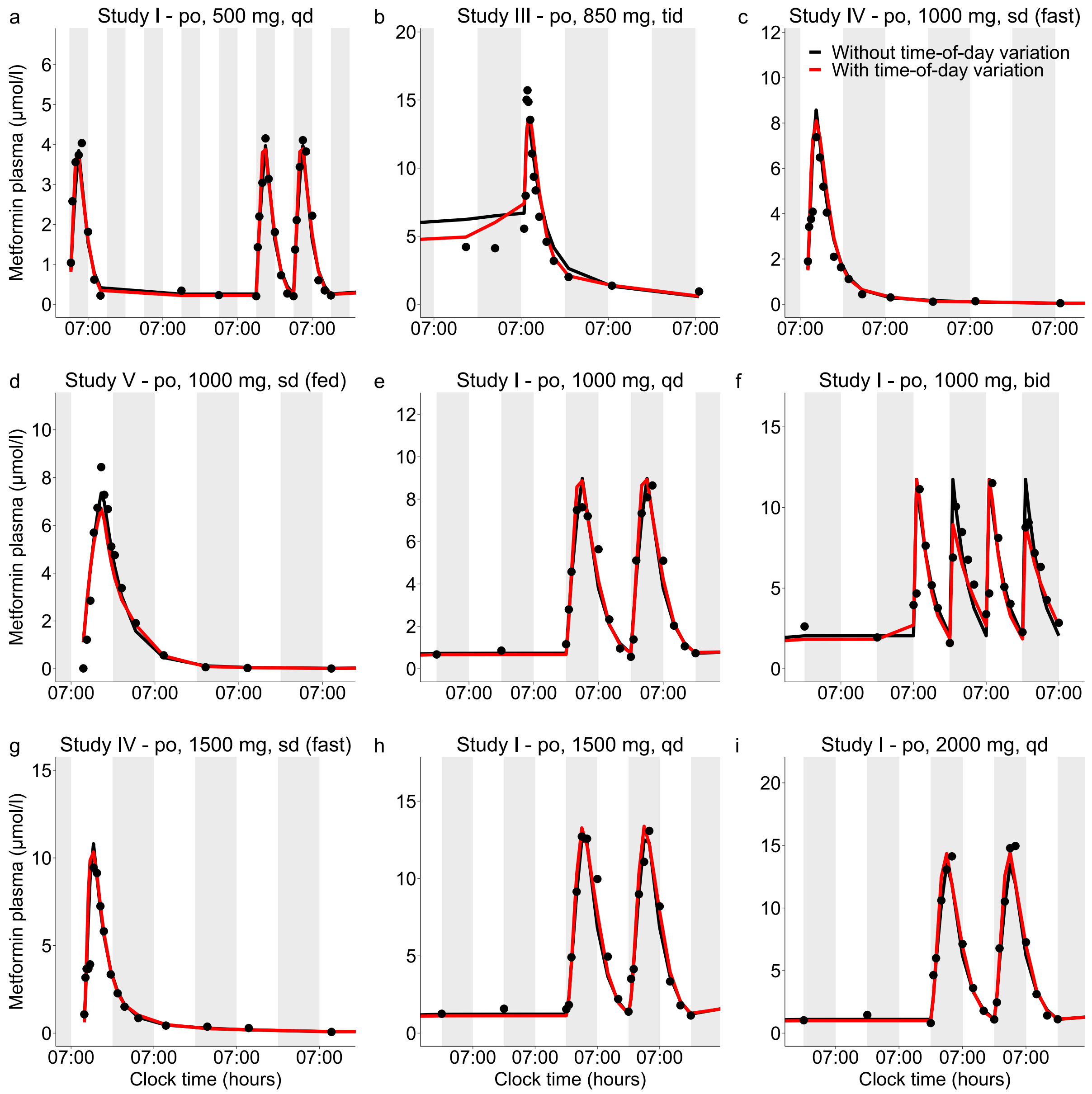

ESM Fig 10. Exemplary individual plasma concentration-time profiles for each administered regimen (linear plots). Metformin was administered as (**a**, **c**, **d**, **e**, **g**, **h**, **i**) extended-release or (**b**, **f**) immediate-release tablet. Observed data from studies I-V [1–5] are shown as dots, predictions are shown as lines (black: without time-of-day variation, red: with time-of-day variation). Grey areas indicate night-time. bid, twice daily; fast, fasted state; fed, fed state; po, oral; sd, single dose; tid, three times daily; qd, once daily

2.2.1.2 Metformin urine concentration-time profiles

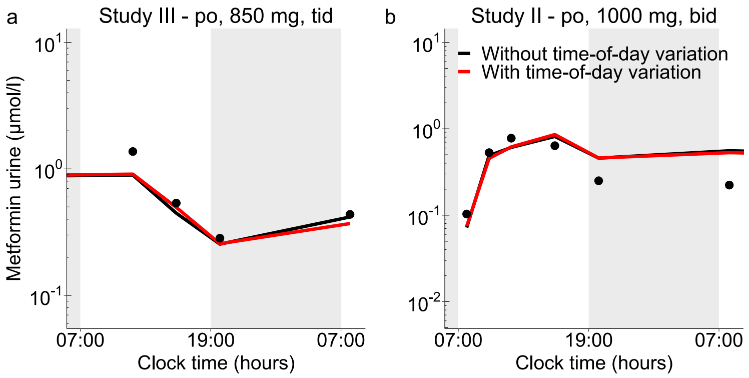

ESM Fig 11. Exemplary individual urine concentration-time profiles (semilogarithmic plots, i.e. concentration presented on decadic logarithm scale). Observed data from studies II and III [2, 3] are shown as dots, predictions are shown as lines (black: without time-of-day variation, red: with time-of-day variation). Grey areas indicate night-time. bid, twice daily; po, oral; tid, three times daily

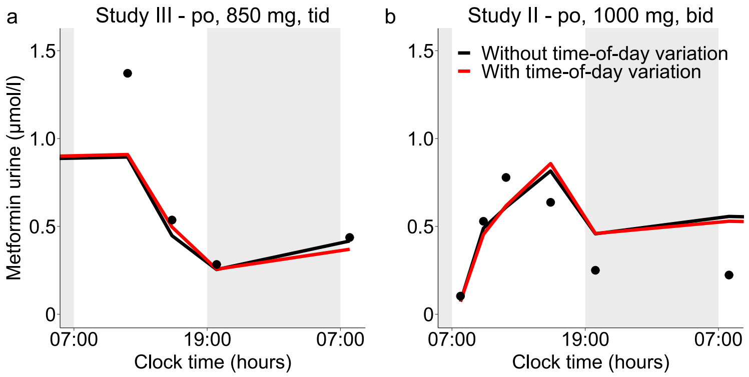

ESM Fig 12. Exemplary individual urine concentration-time profiles (linear plots). Observed data from studies II and III [2, 3] are shown as dots, predictions are shown as lines (black: without time-of-day variation, red: with time-of-day variation). Grey areas indicate night-time. bid, twice daily; po, oral; tid, three times daily

2.2.1.3 Metformin goodness-of-fit plots

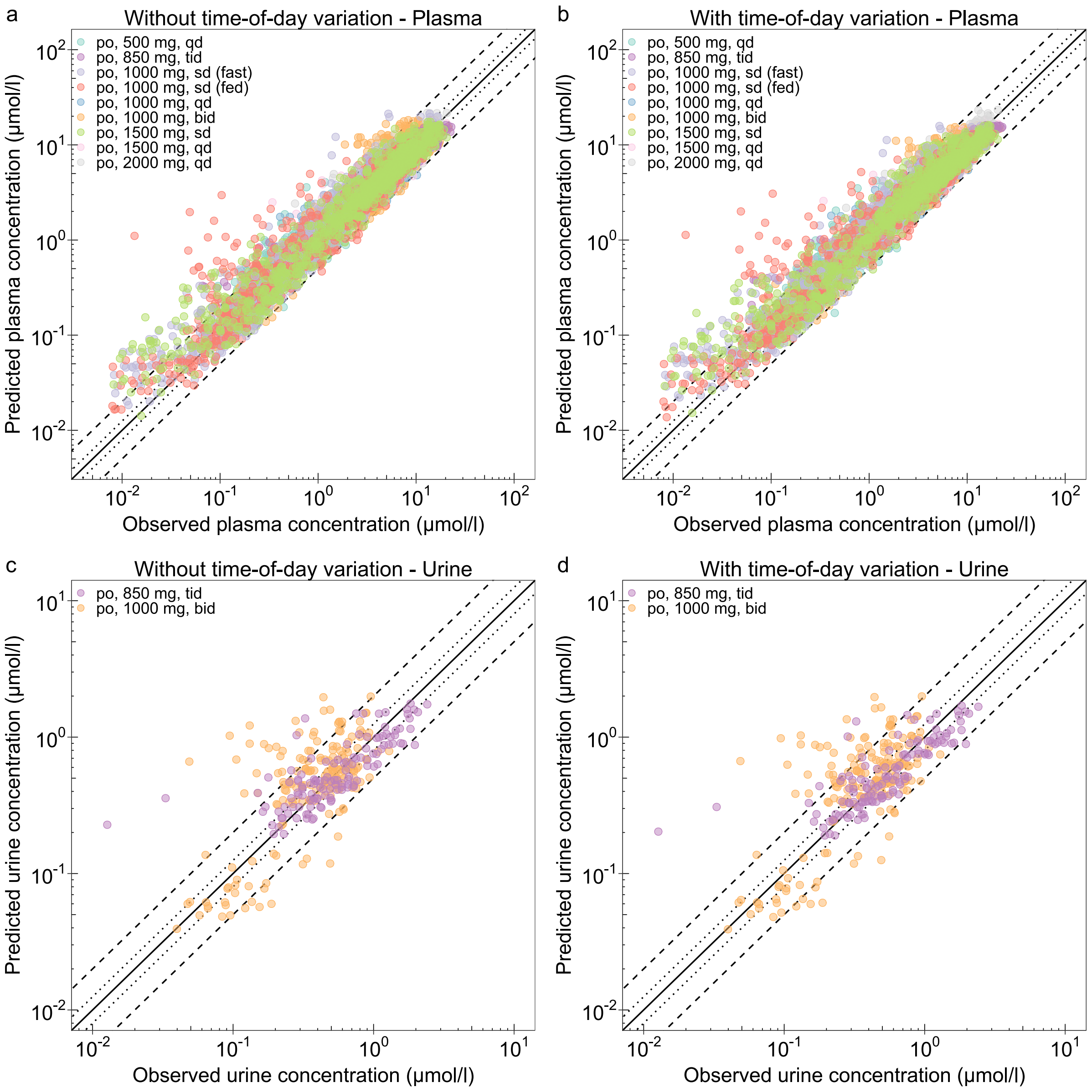

ESM Fig 13. Goodness-of-fit plots, showing NLME pharmacokinetic model predictions compared to observed metformin (**a**—**b**) plasma and (**c**—**d**) urine concentrations of individuals from studies I-V [1–5], receiving either metformin extended- or immediate-release formulations. Predictions are shown for the model (**a**, **c**) without and (**b**, **d**) with time-of-day variation. The straight black line marks the line of identity. Dotted lines indicate 0.8- to 1.25-fold and dashed lines indicate 0.5- to 2-fold acceptance limits. bid, twice daily; fast, fasted state; fed, fed state; po, oral; sd, single dose; tid, three times daily; qd, once daily

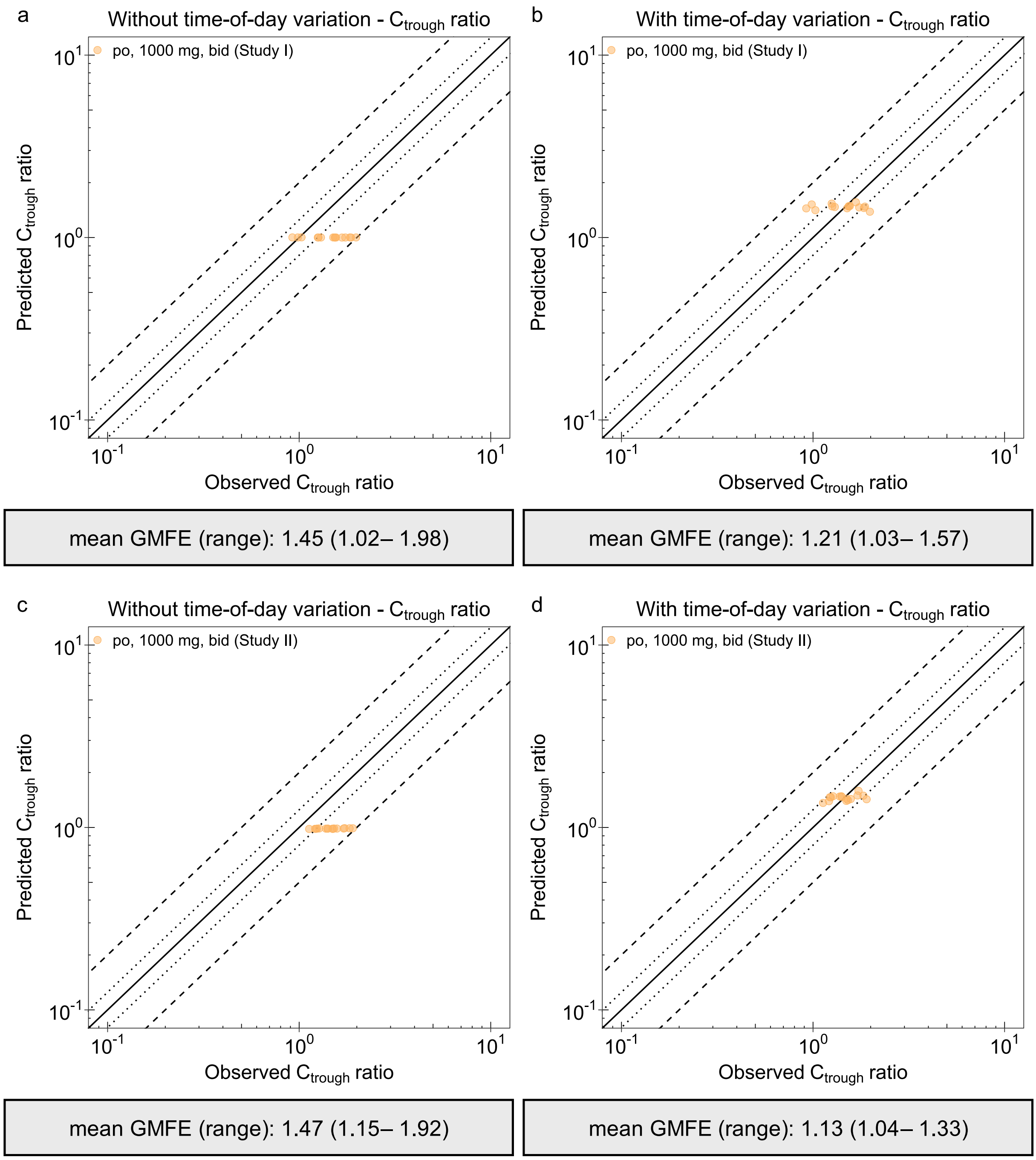

ESM Fig 14. Goodness-of-fit plots, showing NLME pharmacokinetic model predictions compared to observed metformin C_{trough} ratios (morning/evening) from studies I and II [1, 2], receiving twice daily 1000 mg of metformin immediate-release formulation ((**a**–**b**) study I and (**c**–**d**) study II), comparing the model (**a**–**c**) without and (**b**–**d**) with time-of-day variation. The straight black line marks the line of identity. Dotted lines indicate 0.8- to 1.25-fold and dashed lines indicate 0.5- to 2-fold acceptance limits. bid, twice daily; GMFE, geometric mean fold error; po, oral

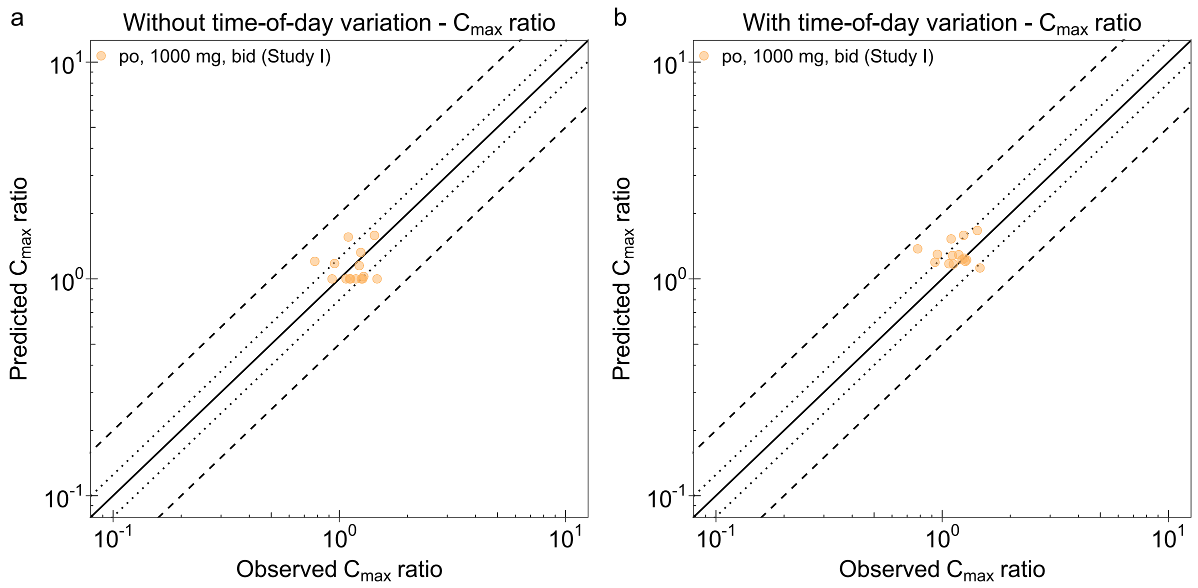

mean GMFE (range): 1.21 (1.06– 1.55)	mean GMFE (range): 1.19 (1.00– 1.77)

ESM Fig 15. Goodness-of-fit plots, showing NLME pharmacokinetic model predictions compared to observed metformin C_{max} ratios (morning/evening) from studies I and II [1, 2], receiving twice daily 1000 mg of metformin immediate-release formulation (study I), comparing the model (**a**) without and (**b**) with time-of-day variation. The straight black line marks the line of identity. Dotted lines indicate 0.8- to 1.25-fold and dashed lines indicate 0.5- to 2-fold acceptance limits. bid, twice daily; GMFE, geometric mean fold error; po, oral

2.2.1.4 Conditional weighted residuals vs. time

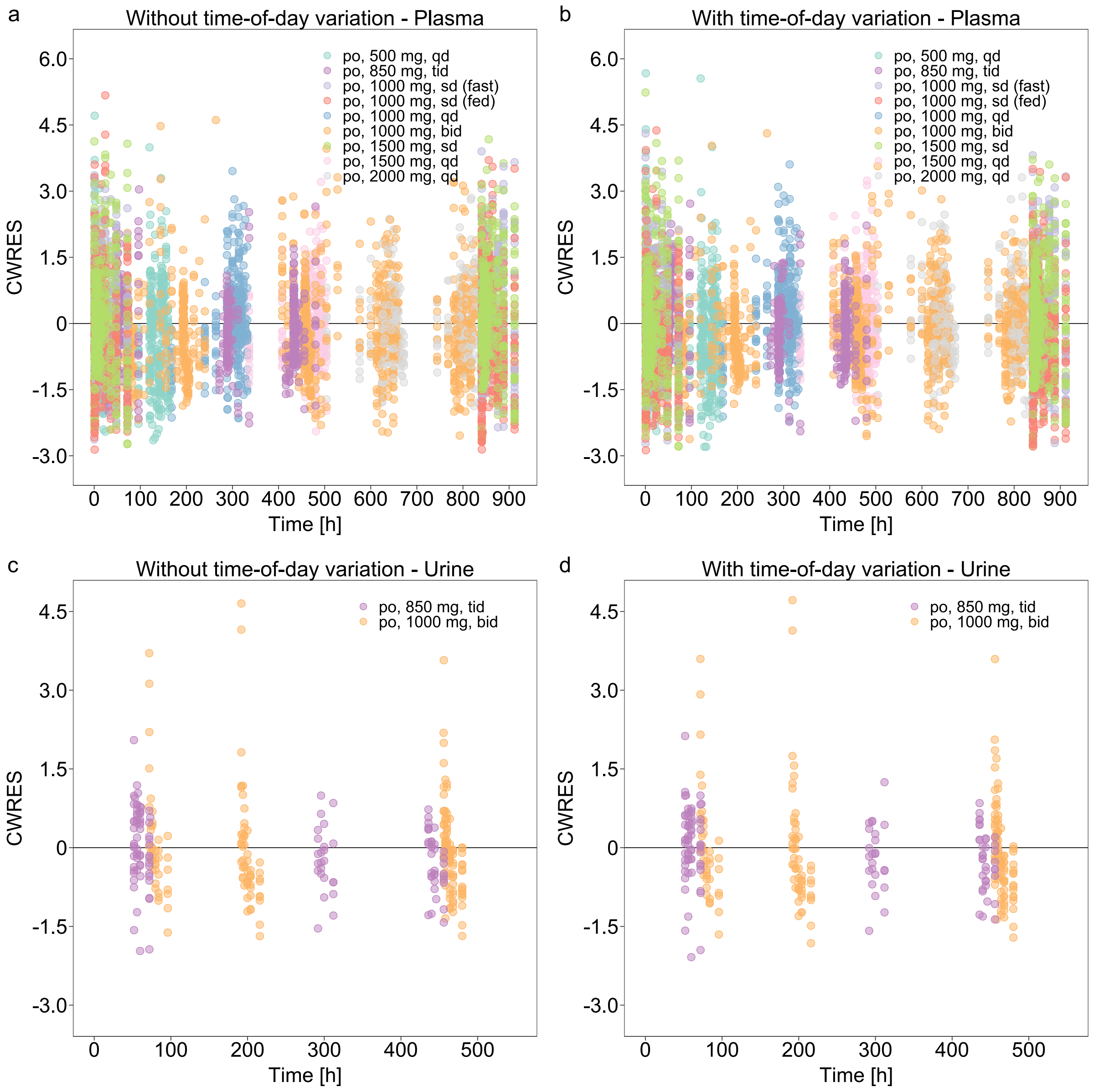

ESM Fig 16. NLME pharmacokinetic model conditional weighted residuals (CWRES) vs. time for the model (**a**, **c**) without and (**b**, **d**) with time-of-day variation taking (**a**—**b**) metformin plasma concentrations and (**c**—**d**) metformin urine concentrations from studies I-V [1–5] into account. bid, twice daily; fast, fasted state; fed, fed state; po, oral; sd, single dose; tid, three times daily; qd, once daily

2.2.1.5 Conditional weighted residuals vs. prediction

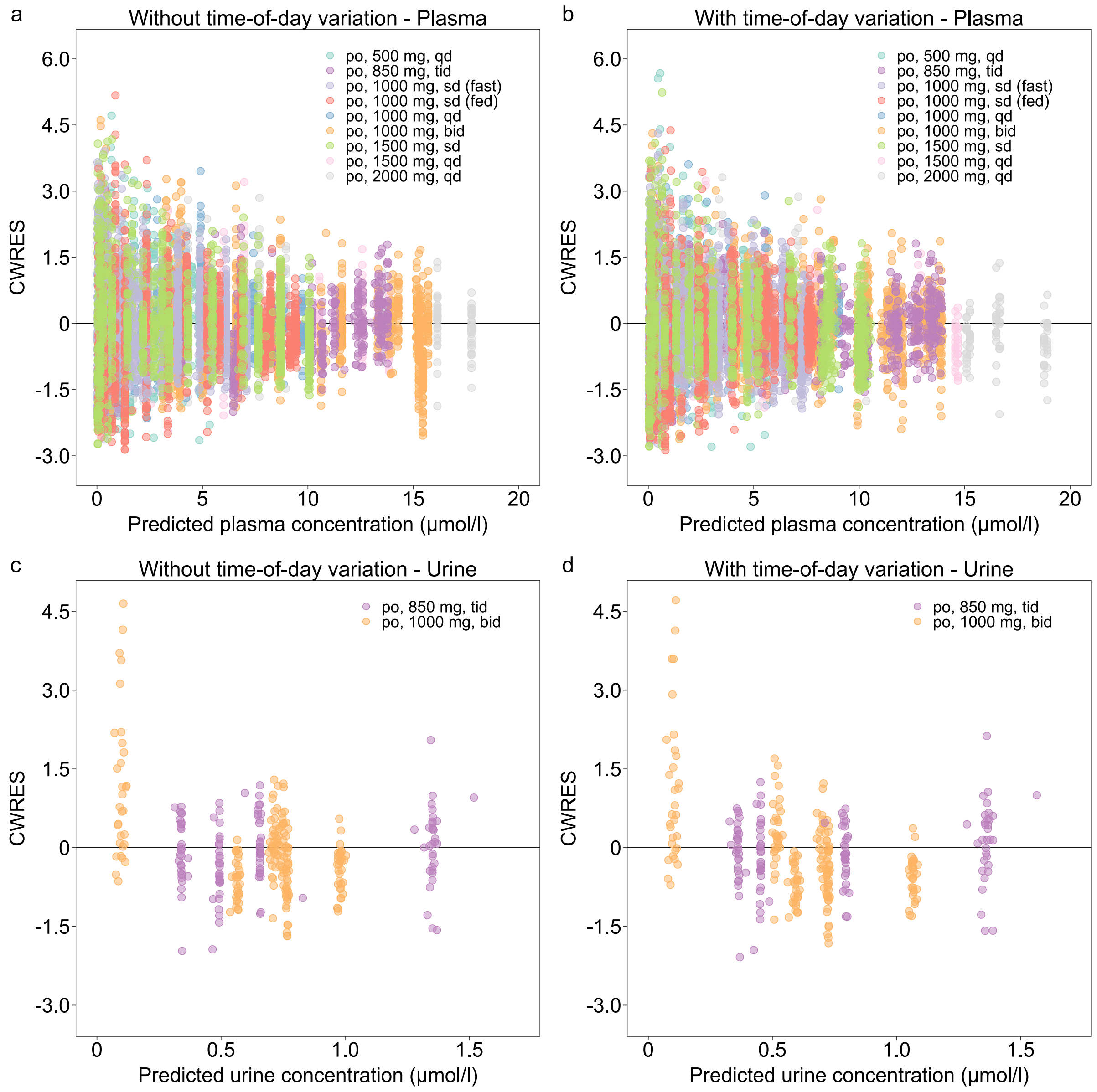

ESM Fig 17. NLME pharmacokinetic model conditional weighted residuals (CWRES) vs. prediction for the model (**a**, **c**) without and (**b**, **d**) with time-of-day variation taking (**a**—**b**) metformin plasma concentrations and (**c**—**d**) metformin urine concentrations from studies I-V [1–5] into account. bid, twice daily; fast, fasted state; fed, fed state; po, oral; sd, single dose; tid, three times daily; qd, once daily

2.3 Literature-informed mechanistic PBPK modelling

2.3.1 Literature search

ESM Table 8. Time-of-day variation of pharmacokinetic-related processes and physiological conditions according to Dallmann et al. [20]

ADME process	Amplitude (%)	Acrophase (Clock time, hours)	Reference
Absorption			
Gastric pH	35	09:00	[21]
Gastric emptying time	20 (solid), 7 (liquid)[a]	20:00[b]	[22]
Gut motility	56	14:00	[23]
Blood flow to GIT	15	04:00	[24]
Distribution			
Hepatic blood flow	15	04:00	[24]
Excretion			
Glomerular filtration rate	13	15:01	[25–27]
Renal plasma flow	12	17:22	[25–27]

[a] Amplitudes calculated for emptying half-times, [b] meals were given at 08:00 and 20:00 hours. GIT, gastrointestinal tract

2.3.2 Hypothesis testing

Daily variation of processes and physiological conditions identified during literature search were rated quantitatively (ESM Fig 18) and graphically regarding prediction accuracy (ESM Fig 19).

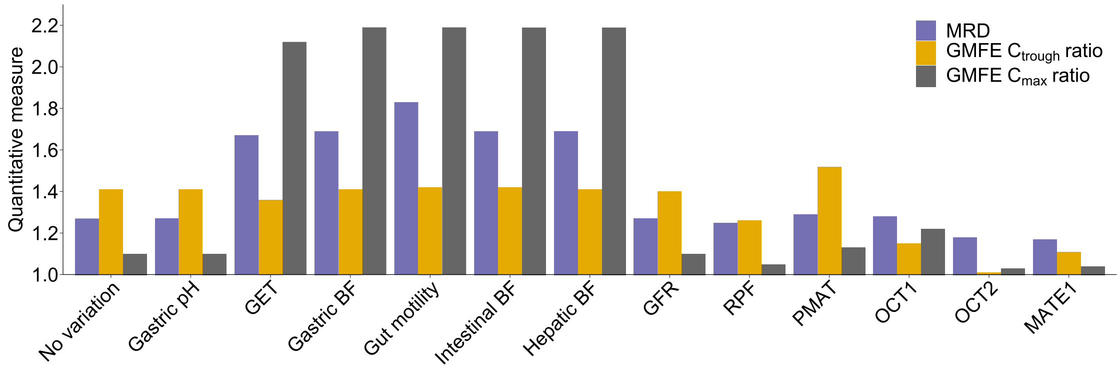

ADME process	MRD	GMFE C_{trough} ratio	GMFE C_{max} ratio
No time-of-day variation	1.27	1.41	1.10
Absorption			
Gastric pH (lit.)	1.27	1.41	1.10
Gastric emptying time (lit.)	1.67	1.36	2.12
Gastric blood flow (lit.)	1.69	1.41	2.19
Gut motility (lit.)	1.83	1.42	2.19
Intestinal blood flow (lit.)	1.69	1.42	2.19
Distribution			
Hepatic blood flow (lit.)	1.69	1.41	2.19
Excretion			
Glomerular filtration rate (lit.)	1.27	1.40	1.10
Renal plasma flow (lit.)	1.25	1.26	1.05
Transporter			
PMAT (opt.)	1.29	1.52	1.13
OCT1 (opt.)	1.28	1.15	1.22
OCT2 (opt.)	1.18	1.01	1.03
MATE1 (opt.)	1.17	1.11	1.04

ESM Fig 18. Hypothesis testing with the PBPK model, assuming rhythmic physiological processes. BF, blood flow; C_{max}, maximum plasma concentration; C_{trough}, trough plasma concentration; GET, gastric emptying time; GMFE, geometric mean fold error; lit, literature; MATE, multidrug and toxin extrusion protein; MRD, mean relative deviation; OCT, organic cation transporter; opt, optimised; PMAT, plasma membrane monoamine transporter; RPF, renal plasma flow

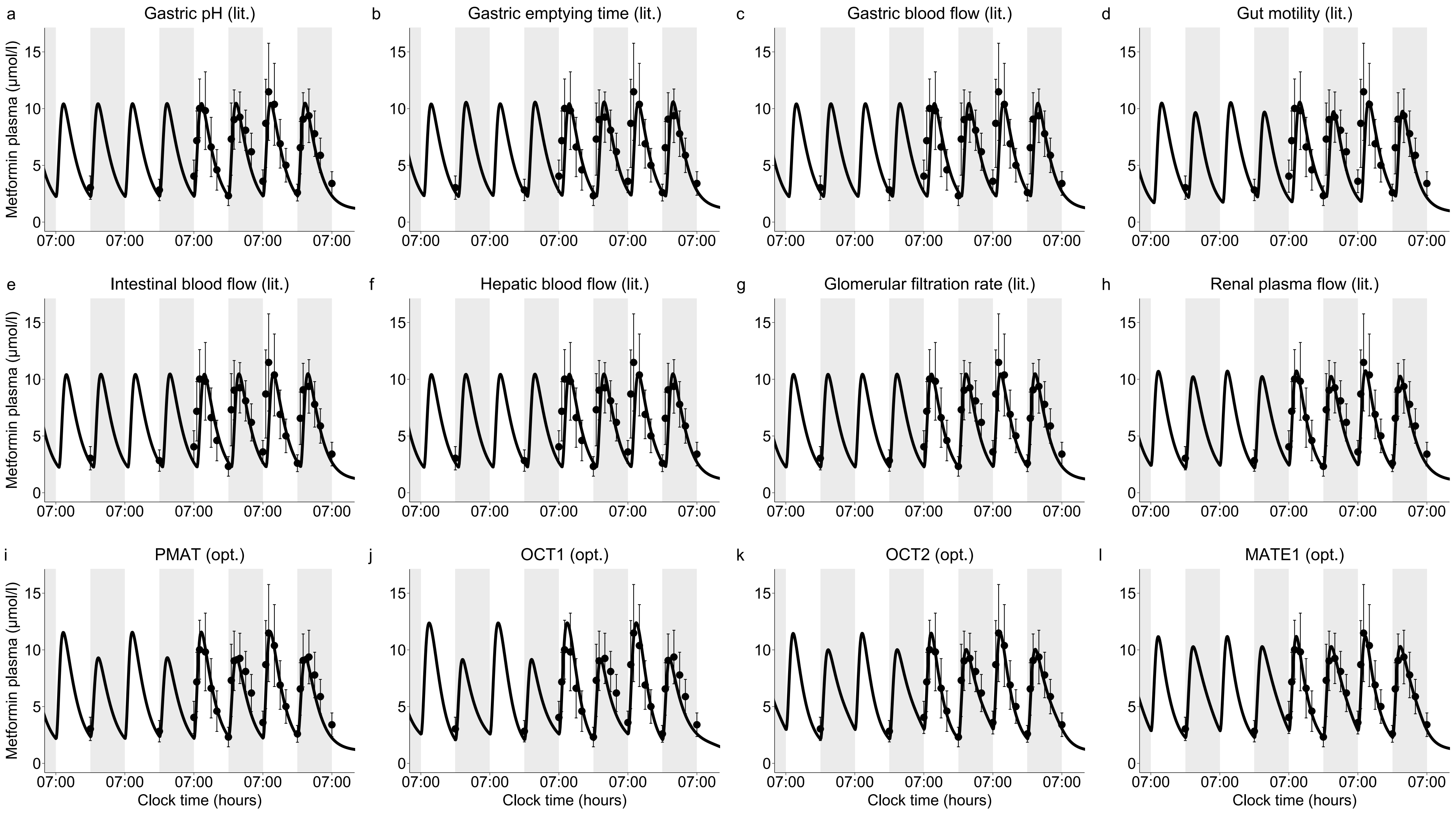

ESM Fig 19. PBPK model predictions alongside observed data of study I [1] (1000 mg twice daily immediate-release administration in the fed state), assuming a daily oscillation of different processes and physiological conditions. Observed data are shown as dots ± SD, predictions are shown as lines. Grey areas indicate night-time. lit, literature; MATE, multidrug and toxin extrusion protein; OCT, organic cation transporter; opt, optimised; PMAT, plasma membrane monoamine transporter

2.3.3 Final PBPK model parameters (with daily oscillation)

Individual plasma concentration-time profiles show high interindividual variability. Therefore, organic cation transporter (OCT) 2 transport rate constant (k_{cat}) values were optimised for every individual. Additionally, OCT2 amplitude and time shift were optimised (after inclusion of rhythmic GFR and RPF) with the help of individual profiles (*n*=26) (ESM Table 10). A correlation plot of OCT2 k_{cat} values, amplitude and time shift (sine function describing daily oscillation) is shown in ESM Fig 20, where no correlation has been detected.

ESM Table 9. Drug-dependent parameters of the metformin PBPK model adopted from Hanke et al.[8]

Parameter	Value	Unit	Source	Literature	Reference	Description
MW	129.16	g/mol	Literature	129.16	[28]	Molecular weight
pKa_1 (base)	2.80		Literature	2.80	[29]	Acid dissociation constant
pKa_2 (base)	11.50		Literature	11.50	[29]	Acid dissociation constant
Solubility (pH 6.8)	350.90	g/l	Literature	350.90	[29]	Solubility
logP	-1.43		Literature	-1.43	[30]	Lipophilicity
fu	100	%	Literature	100	[31–33]	Fraction unbound plasma
B/P ratio	-		Optimised	Time-dependent	[31]	Blood/plasma concentration ratio
MATE1 K_M	283.00	µmol/l	Literature	283.00	[34]	Michaelis-Menten constant
MATE1 k_{cat}	165.69	1/min	Optimised	-	-	Transport rate constant
OCT1 K_M	1180.00	µmol/l	Literature	1180.00	[35]	Michaelis-Menten constant
OCT1 k_{cat}	641.19	1/min	Optimised	-	-	Transport rate constant
OCT2 K_M	810.00	µmol/l	Literature	810.00	[35]	Michaelis-Menten constant
OCT2 k_{cat}	5.17×10^4	1/min	Optimised	-	-	Transport rate constant
PMAT K_M	367.57	µmol/l	Optimised	1320.00	[36]	Michaelis-Menten constant
PMAT k_{cat}	76.47	1/min	Optimised	-	-	Transport rate constant
PMAT Hill	3.00		Literature	2.64	[36]	Hill coefficient
GFR fraction	1		Assumed	-	-	Fraction of filtered drug in the urine
EHC continuous fraction	1		Assumed	-	-	Fraction of bile continually released
Partition coefficients	Diverse		Calculated	PK-Sim	[37]	Cell to plasma partition coefficients
Cellular permeability	2.30×10^{-4}	cm/min	Calculated	CDS norm.	[38]	Plasma permeability into the cellular space
Intestinal permeability	8.49×10^{-7}	cm/min	Optimised	1.87×10^{-7}	Calculated	Transcellular intestinal permeability
Basolat. small intest. permeability	1.16×10^{-5}	cm/min	Optimised	1.11×10^{-6}	Calculated	Basolateral permeability out of the mucosa
Basolat. large intest. permeability	0	cm/min	Assumed	1.11×10^{-6}	Calculated	Basolateral permeability out of the mucosa
Formulation	IR fast/IR fed[a]		Optimised	-	[8, 39, 40]	Formulation used in predictions

[a] IR fast: Weibull function with a dissolution time of 7.90 minutes and a dissolution shape of 1.36 (extracted from [39]), IR fed: Weibull function with a dissolution time of 7.90 minutes and a dissolution shape of 0.11 (both optimised) [8, 40], ER fed: Weibull function with a dissolution time of 402.80 minuted and a dissolution shape of 1.35 (both optimised) [1, 41, 42]. basolat., basolateral; CDS, norm. charge-dependent Schmitt normalised to PK-Sim calculation method; EHC, enterohepatic circulation; ER, extended-release formulation; intest., intestinal; IR, immediate-release formulation; MATE, multidrug and toxin extrusion protein; OCT, organic cation transporter; PK-Sim, PK-Sim standard calculation method; PMAT, plasma membrane monoamine transporter

ESM Table 10. System- and drug-dependent PBPK model parameters to cover time-of-day dependent and interindividual variability

Parameter	Value		Unit	Source	Literature	Reference	Description
	mean	range					
GFR amplitude	13	-	%	Literature	13	[25–27]	Amplitude sine function
GFR acrophase	15:01	-	Clock time, hours	Literature	15:01	[25–27]	Acrophase sine function
RPF amplitude	12	-	%	Literature	12	[25–27]	Amplitude sine function
RPF acrophase	17:22	-	Clock time, hours	Literature	17:22	[25–27]	Acrophase sine function
OCT2 k_{cat}	5.77×10^4	1.29×10^4–1.68×10^5	1/min	Optimised	-	-	Individual transport rate constant
OCT2 amplitude	57	11–98	%	Optimised	-	-	Individual amplitude sine function
OCT2 acrophase	16:54	12:16 –20:41 hours	Clock time, hours	Optimised	-	-	Individual acrophase sine function

A 24-hour phase was assumed for the sine function. OCT, organic cation transporter; RPF, renal plasma flow

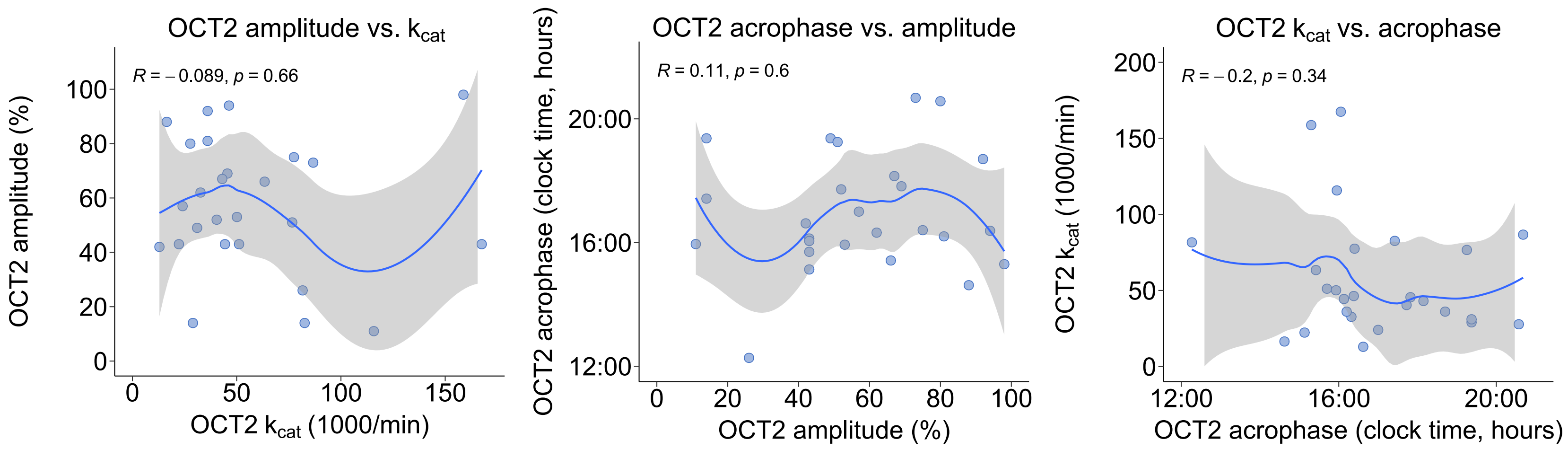

ESM Fig 20. Correlation plots of individual PBPK modelled OCT2 k_{cat}, amplitude and time shift values to cover daily oscillation (n=26). k_{cat}, transport rate constant; OCT, organic cation transporter

2.3.4 PBPK model plots

2.3.4.1 Metformin plasma concentration-time profiles

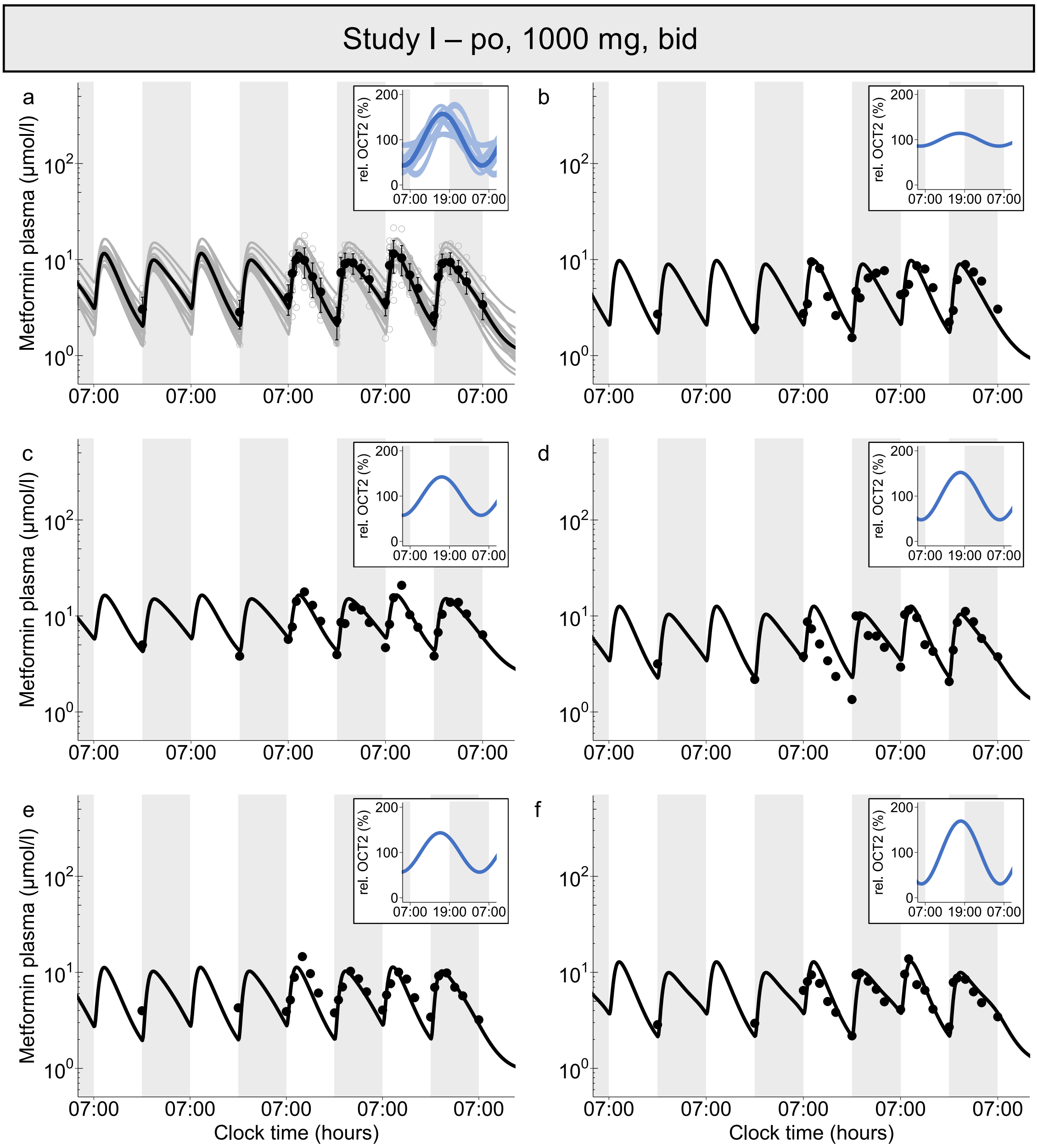

ESM Fig 21. *continued*

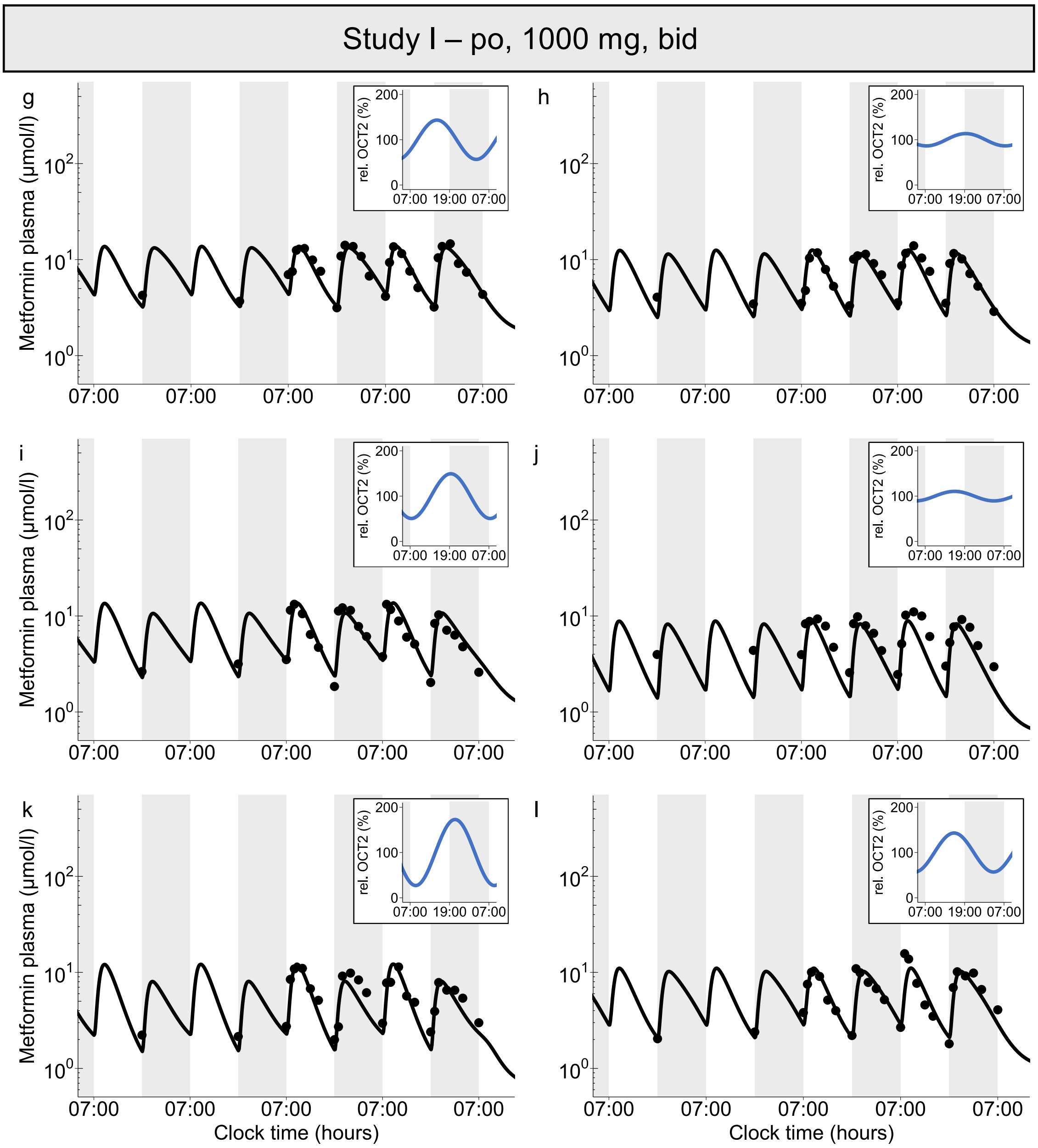

ESM Fig 21. *continued*

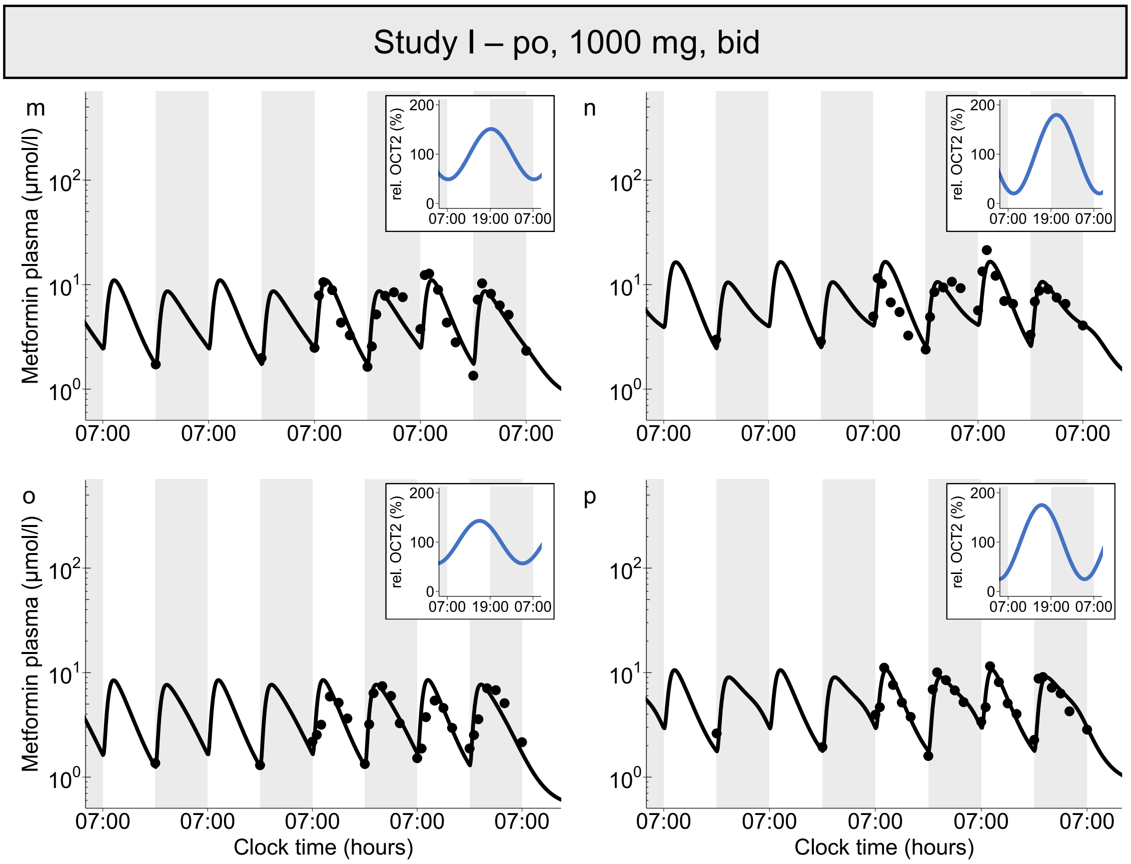

ESM Fig 21. PBPK model predictions compared to observed (**a**) mean and (**b**–**p**) individual plasma concentration-time profiles of metformin after twice daily administration of 1000 mg metformin immediate-release formulation in the fed state (semilogarithmic plots, i.e. concentration presented on decadic logarithm scale, training dataset). Predictions are shown as lines. Observed data from study I are shown as dots ± SD [1]. Grey areas indicate night-time. Inserts depict optimised mean and individual relative OCT2 expression, respectively. bid, twice daily; OCT, organic cation transporter; po, oral

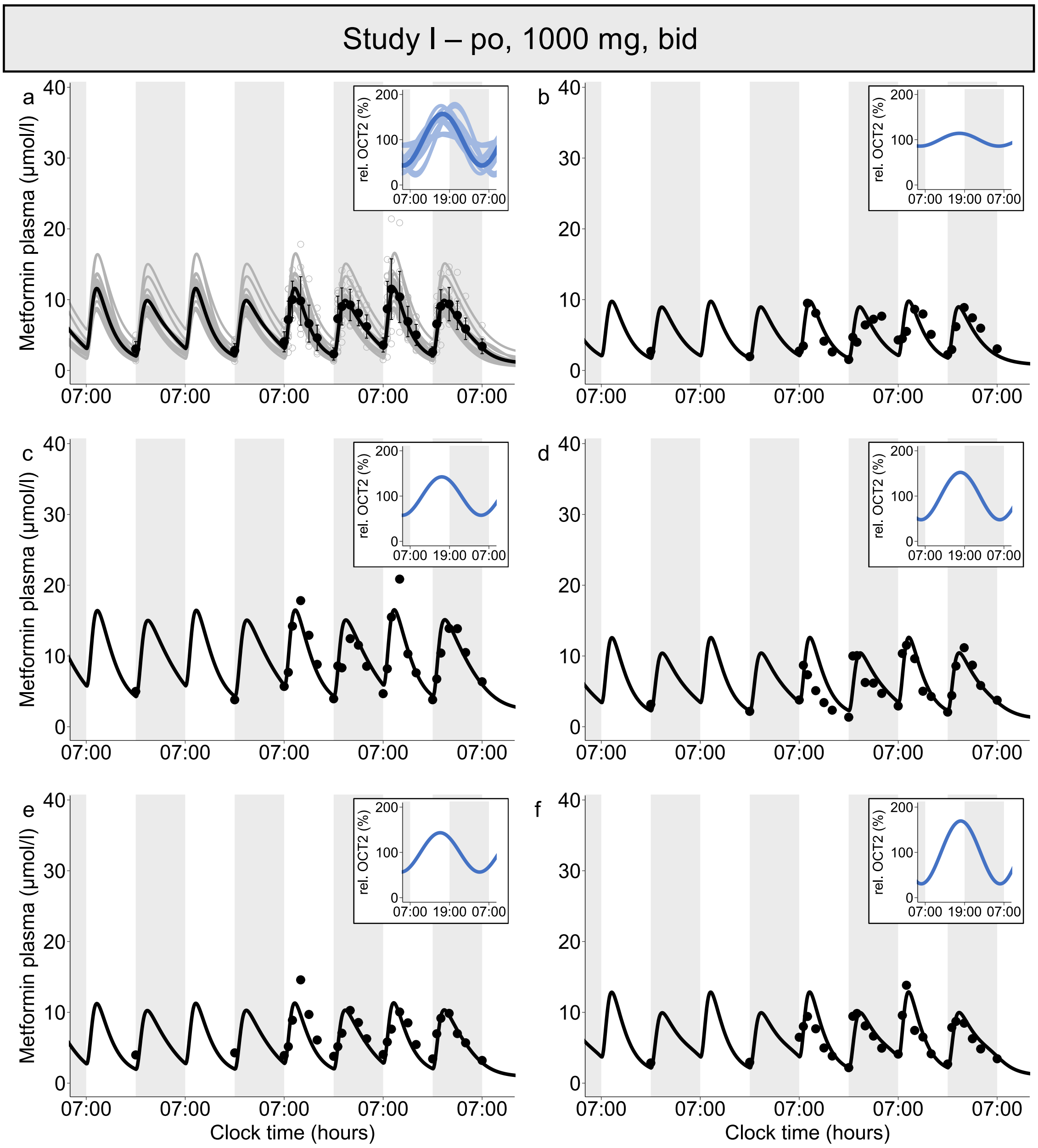

ESM Fig 22. *continued*

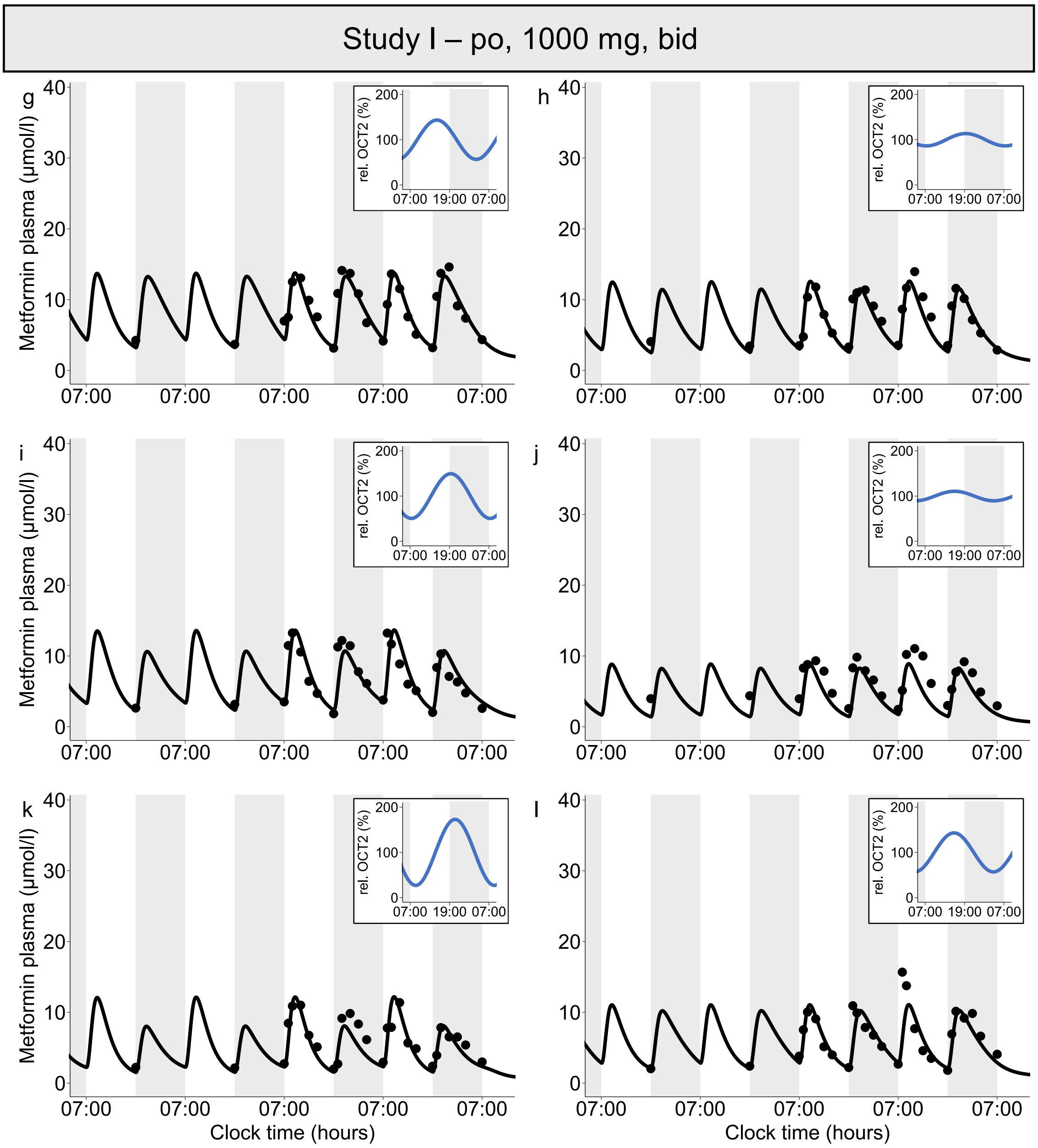

ESM Fig 22. *continued*

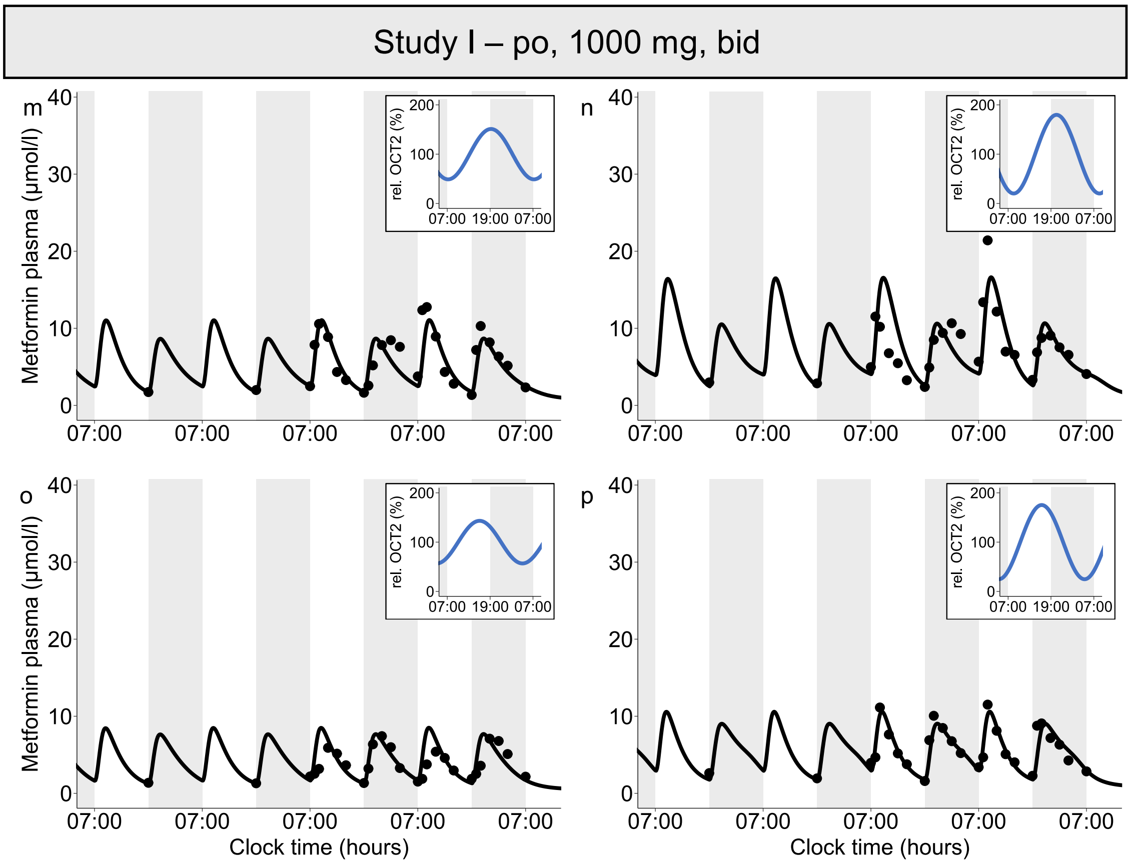

ESM Fig 22. PBPK model predictions compared to observed (**a**) mean and (**b**–**p**) individual plasma concentration-time profiles of metformin after twice daily administration of 1000 mg metformin immediate-release formulation in the fed state (linear plots, training dataset). Predictions are shown as lines. Observed data from study I are shown as dots ± SD [1]. Grey areas indicate night-time. Inserts depict optimised mean and individual relative OCT2 expression, respectively. bid, twice daily; OCT, organic cation transporter; po, oral

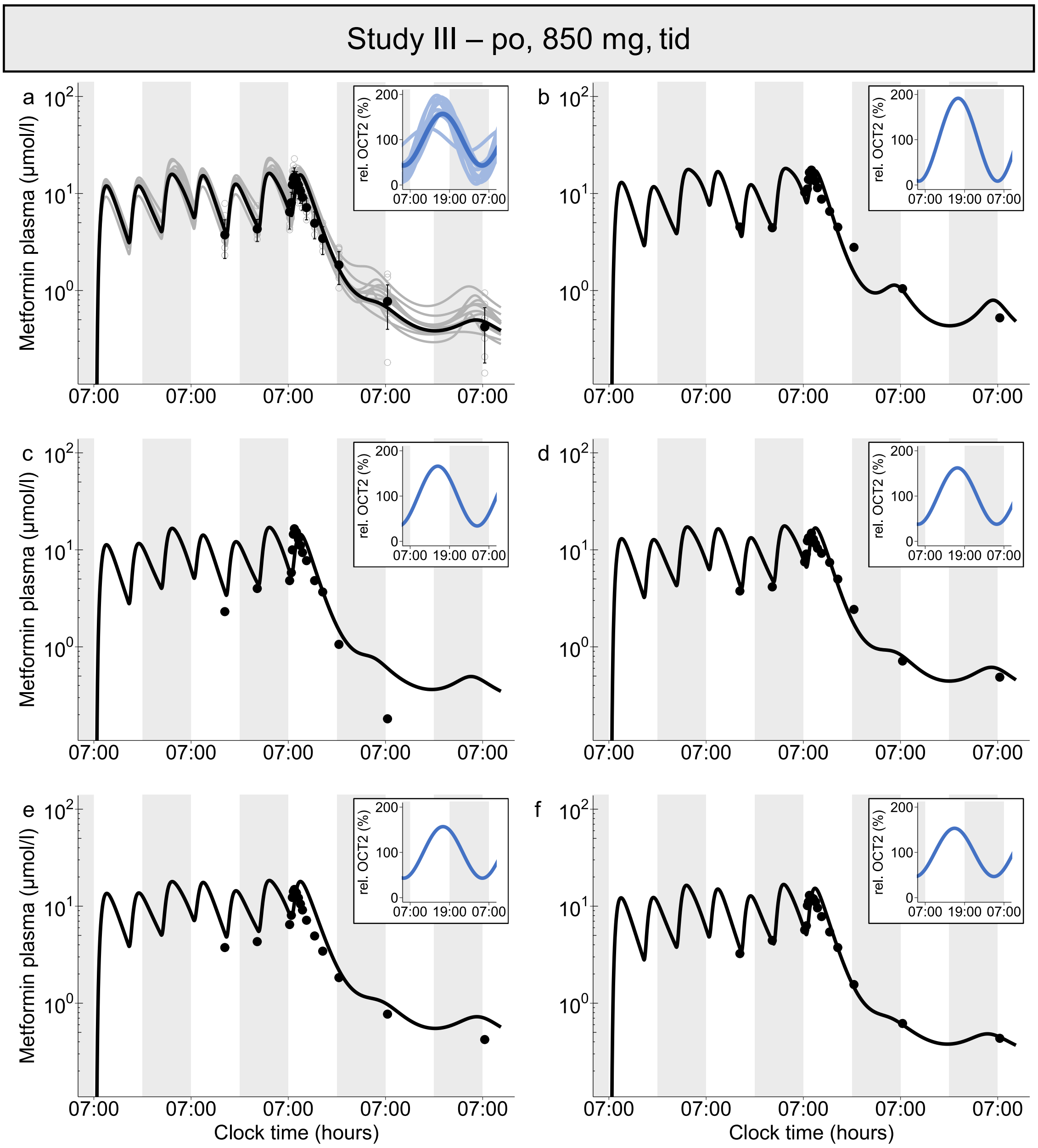

ESM Fig 23. *continued*

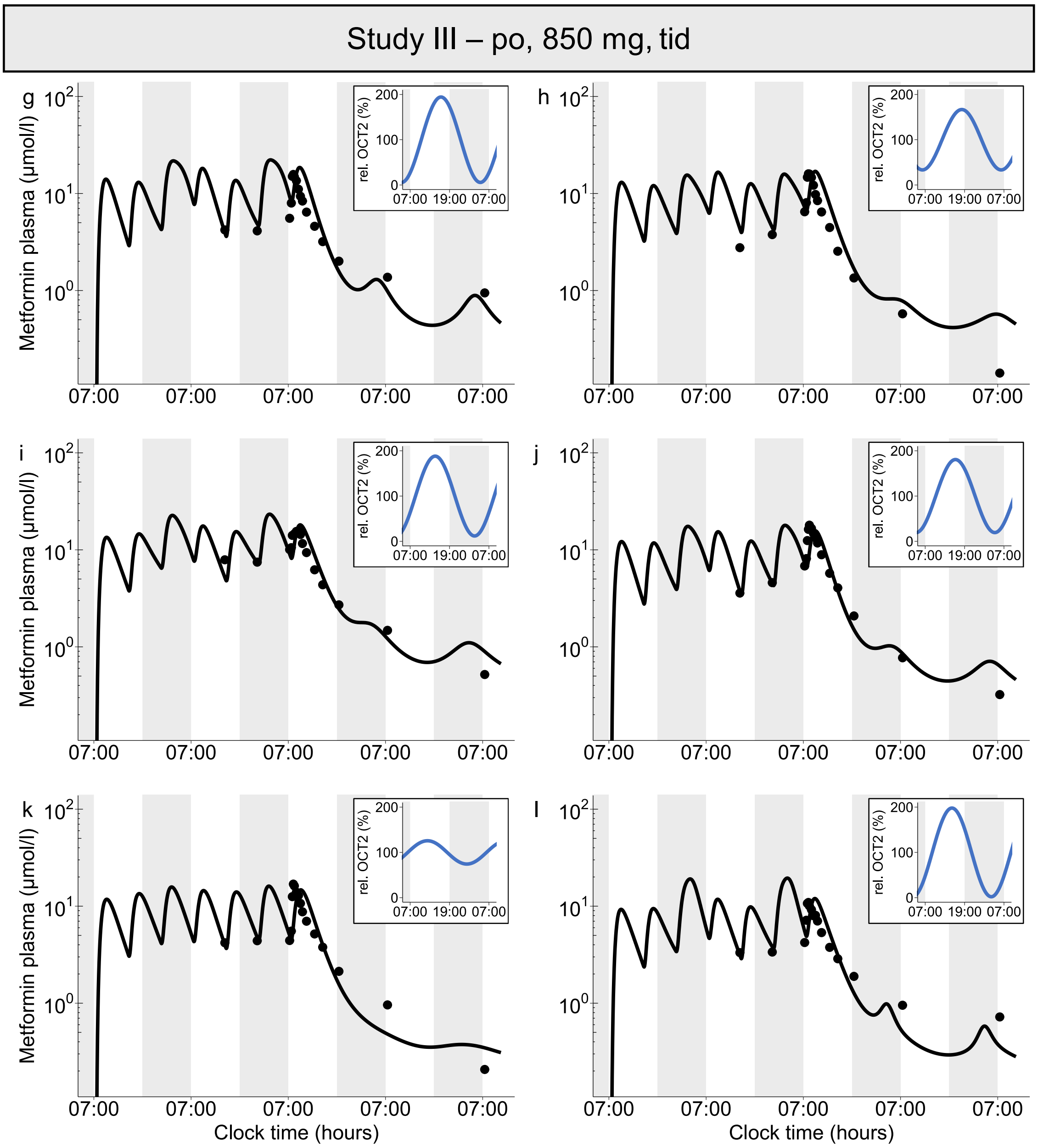

ESM Fig 23. PBPK model predictions compared to observed (**a**) mean and (**b**–**l**) individual plasma concentration-time profiles of metformin after three times daily administration of 850 mg immediate-release formulation (first six doses in fed state, last dose in fasted state) (semilogarithmic plots, i.e. concentration presented on decadic logarithm scale, training dataset). Predictions are shown as lines. Observed data from study III are shown as dots ± SD [3]. Grey areas indicate night-time. Inserts depict optimised mean and individual relative OCT2 expression, respectively. OCT, organic cation transporter; po, oral; tid, three times daily

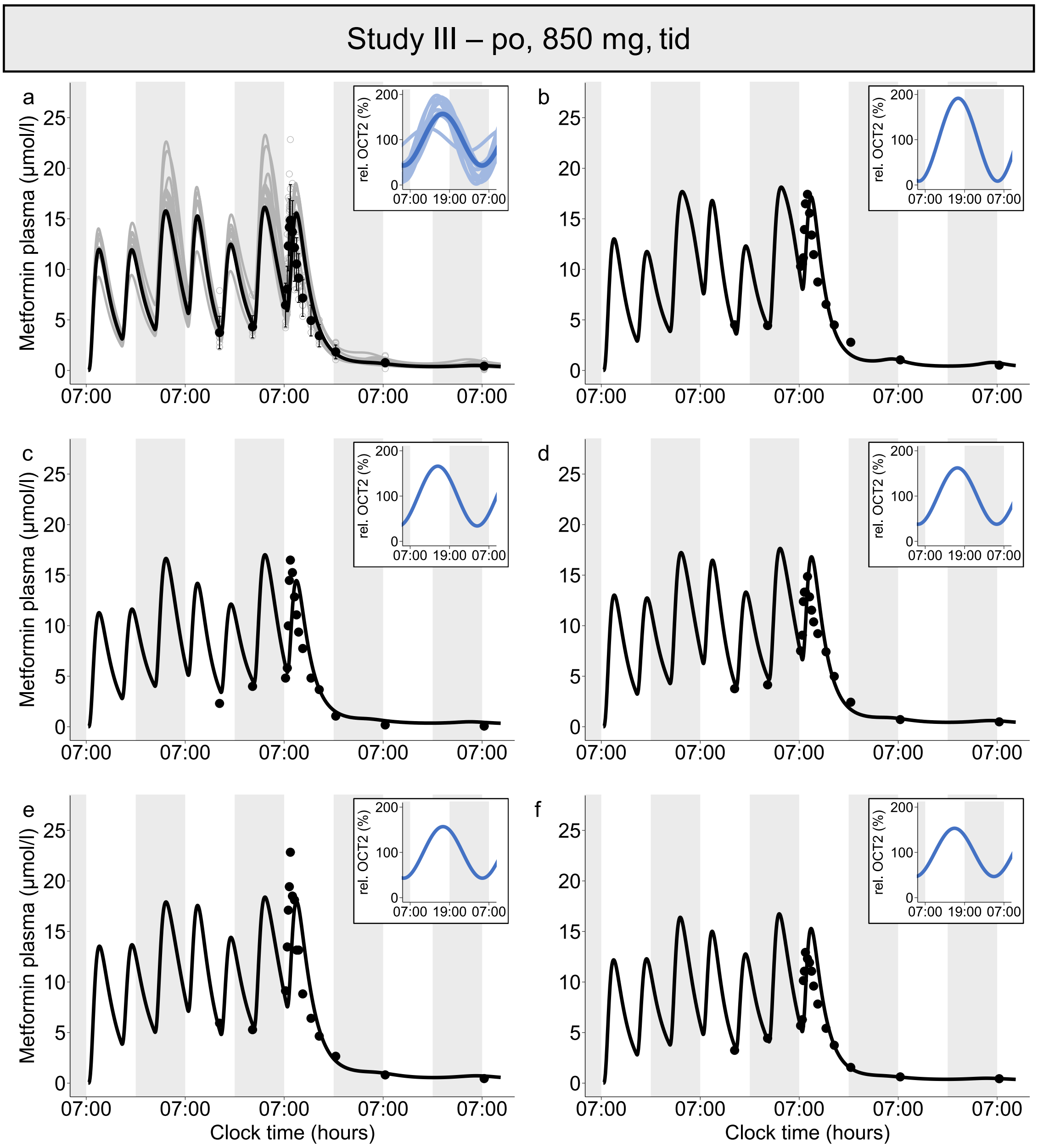

ESM Fig 24. *continued*

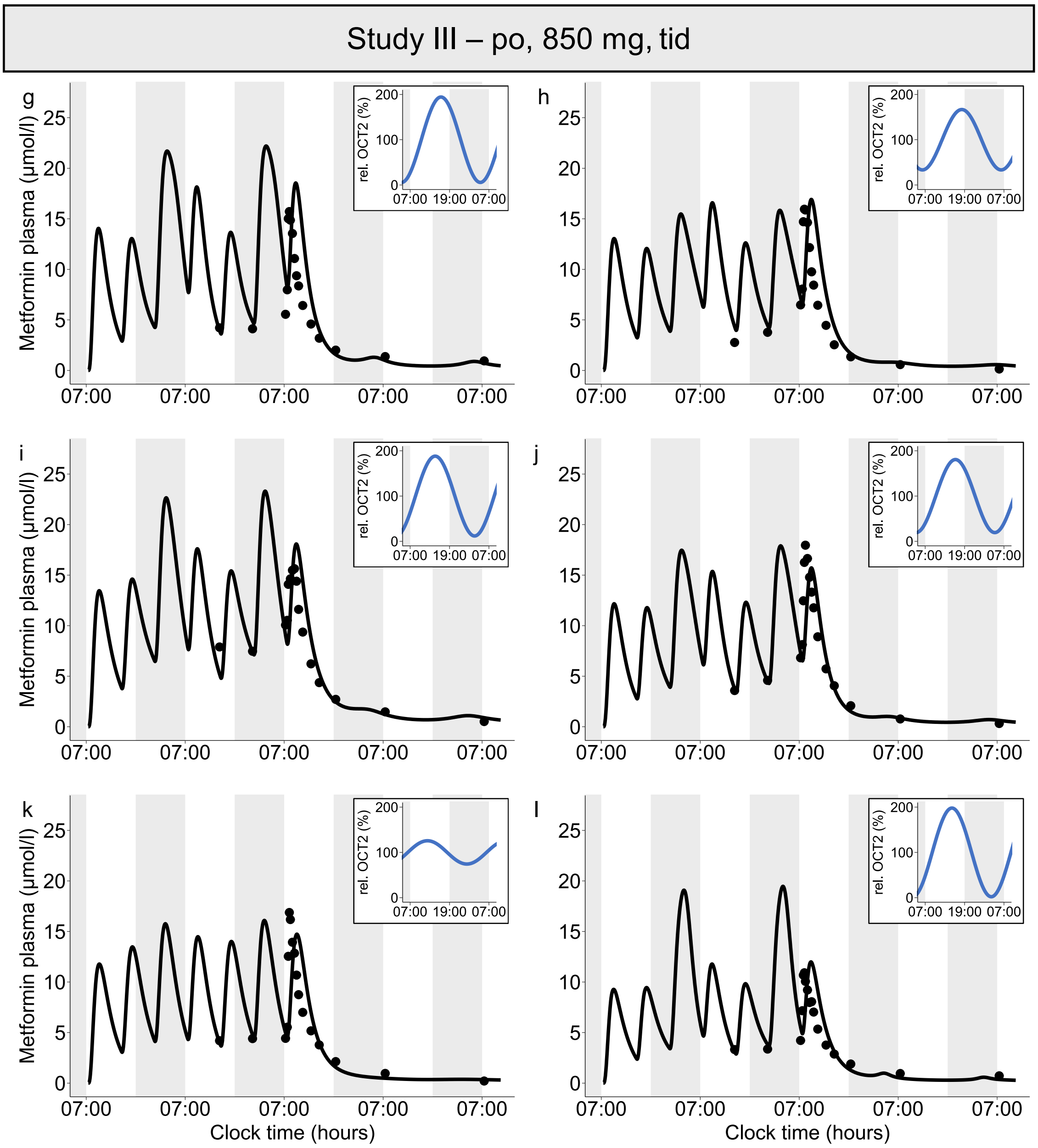

ESM Fig 24. PBPK model predictions compared to observed (**a**) mean and (**b**-**l**) individual plasma concentration-time profiles of metformin after three times daily administration of 850 mg immediate-release formulation (first six doses in fed state, last dose in fasted state) (linear plots, training dataset). Predictions are shown as lines. Observed data from study III are shown as dots ± SD [3]. Grey areas indicate night-time. Inserts depict optimised mean and individual relative OCT2 expression, respectively. OCT, organic cation transporter; po, oral; tid, three times daily

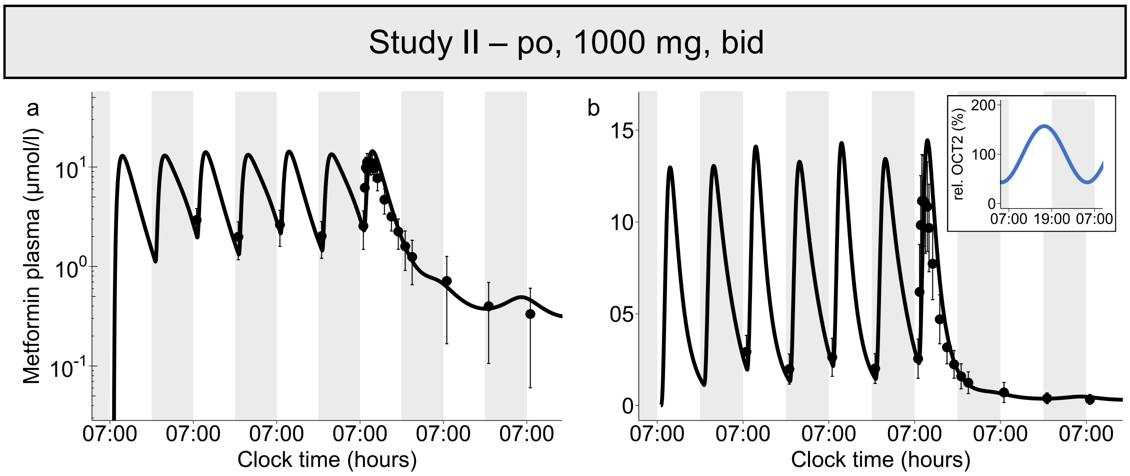

ESM Fig 25. PBPK model predictions compared to observed mean concentration-time profiles of metformin after twice daily administration of 1000 mg metformin immediate-release formulation in the fed state ((**a**) semilogarithmic plot, i.e. concentration presented on decadic logarithm scale, (**b**) linear plot, test dataset). Predictions are shown as lines. Observed data from study II are shown as dots ± SD [2]. Grey areas indicate night-time. Insert depicts optimised mean relative OCT2 expression. bid, twice daily; OCT, organic cation transporter; po, oral

2.3.4.2 Metformin goodness-of-fit plots

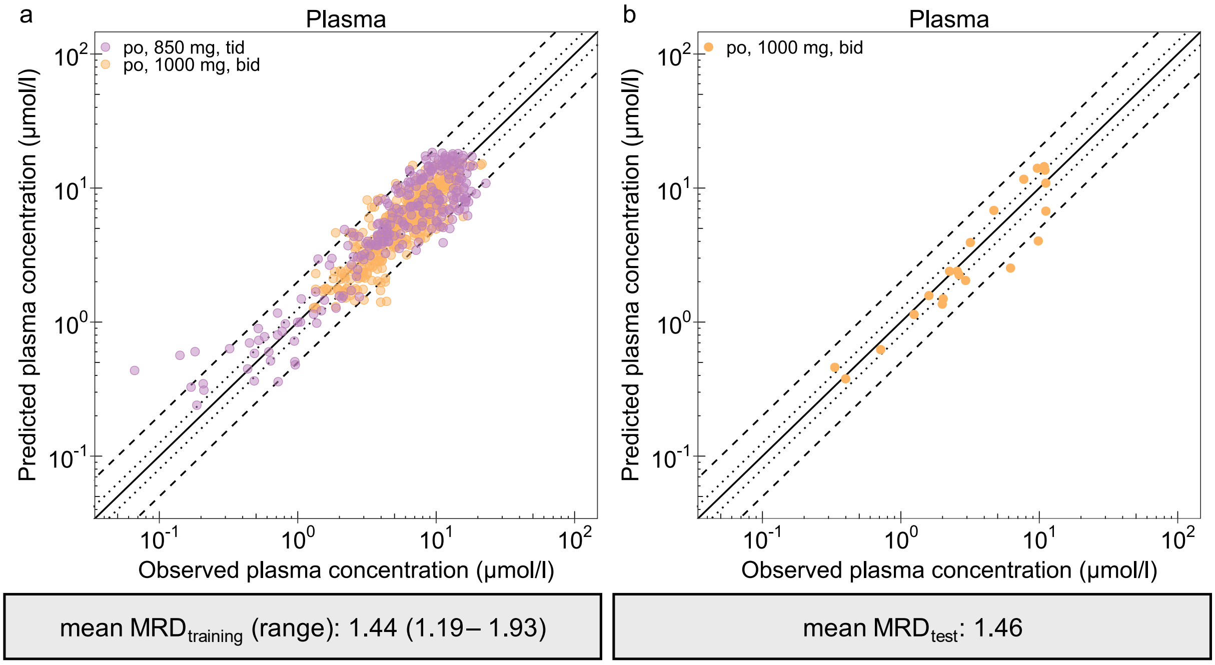

ESM Fig 26. Goodness-of-fit plots, showing PBPK model predictions of (**a**) the training (*n*=26) and (**b**) the test dataset (mean profile) compared to observed metformin plasma concentrations following either twice daily 1000 mg or three times daily 850 mg of metformin immediate-release formulation (study I-III [1–3]). The straight black line marks the line of identity. Dotted lines indicate 0.8- to 1.25-fold and dashed lines indicate 0.5- to 2-fold acceptance limits. bid, twice daily; MRD, mean relative deviation; po, oral; tid, three times daily

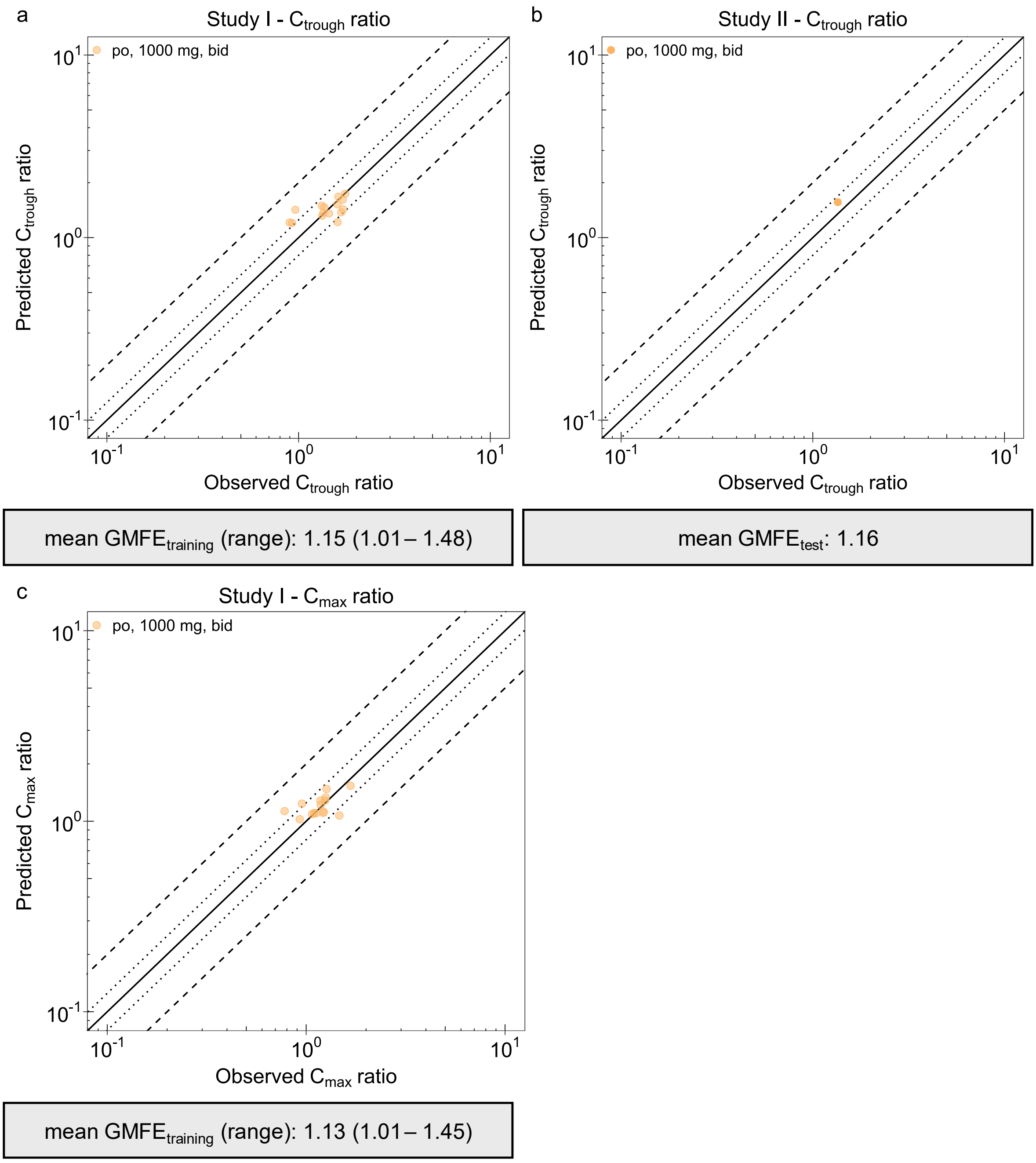

ESM Fig 27. Goodness-of-fit plots, showing PBPK model predictions of (**a**, **c**) the training (*n*=15) and (**b**) the test dataset (mean profile) compared to observed metformin (**a**—**b**) C_{trough} or (**c**) C_{max} ratios (morning/evening), following twice daily 1000 mg of metformin immediate-release formulation (studies I and II [1, 2]). The straight black line marks the line of identity. Dotted lines indicate 0.8- to 1.25-fold and dashed lines indicate 0.5- to 2-fold acceptance limits. bid, twice daily; GMFE, geometric mean fold error; po, oral

3 ESM References

1. Timmins P, Donahue S, Meeker J, Marathe P (2005) Steady-state pharmacokinetics of a novel extended-release metformin formulation. Clin Pharmacokinet 44(7):721–729. https://doi.org/10.2165/00003088-200544070-00004
2. Boehringer Ingelheim Pharma GmbH & Co. KG (2014) Relative Bioavailability BI 10773 and Metformin in Healthy Male Volunteers. ClinicalTrials.gov Identifier: NCT02172248. National Library of Medicine, Bethesda MD; https://clinicaltrials.gov/ct2/show/NCT02172248. Accessed 13 Sep 2022
3. Boehringer Ingelheim Pharma GmbH & Co. KG (2014) Bioavailability of BI 1356 BS and Metformin After Co-administration Compared to the Bioavailability of BI 1356 BS Alone and Metformin Alone in Healthy Male Volunteers. ClinicalTrials.gov Identifier: NCT02183506. National Library of Medicine, Bethesda MD; https://clinicaltrials.gov/ct2/show/NCT02183506. Accessed 13 Sep 2022
4. Boehringer Ingelheim Pharma GmbH & Co. KG (2013) Relative Bioavailability of 2 Fixed Dose Combinations of Linagliptin/Metformin Compared With Single Tablets. ClinicalTrials.gov Identifier: NCT01845077. National Library of Medicine, Bethesda MD; https://www.clinicaltrials.gov/ct2/show/NCT01845077. Accessed 13 Sep 2022
5. Boehringer Ingelheim Pharma GmbH & Co. KG (2013) Relative Bioavailability of 2 Fixed Dose Combinations of Empagliflozin/Metformin Compared With Single Tablets. ClinicalTrials.gov Identifier: NCT01975220. National Library of Medicine, Bethesda MD; https://clinicaltrials.gov/ct2/show/NCT01975220. Accessed 13 Sep 2022
6. Akaike H (1974) A new look at the statistical model identification. IEEE Trans Automat Contr 19(6):716–723. https://doi.org/10.1109/TAC.1974.1100705
7. Beal S, Sheiner L (1998) NONMEM Users Guides. San Francisco: NONMEM Project Group University of California
8. Hanke N, Türk D, Selzer D, et al (2020) A comprehensive whole-body physiologically based pharmacokinetic drug–drug–gene interaction model of metformin and cimetidine in healthy adults and renally impaired individuals. Clin Pharmacokinet 59(11):1419–1431. https://doi.org/10.1007/s40262-020-00896-w
9. Open Systems Pharmacology Suite Community. (2018) PK-Sim® Ontogeny Database Documentation, Version 7.3. https://github.com/Open-Systems-Pharmacology/OSPSuite.Documentation/blob/master/PK-Sim Ontogeny Database Version 7.3.pdf. Accessed 24 Aug 2020
10. Scotcher D, Billington S, Brown J, et al (2017) Microsomal and cytosolic scaling factors in dog and human kidney cortex and application for in vitro-in vivo extrapolation of renal metabolic clearance. Drug Metab Dispos 45(5):556–568. https://doi.org/10.1124/dmd.117.075242
11. Prasad B, Johnson K, Billington S, et al (2016) Abundance of drug transporters in the human kidney

cortex as quantified by quantitative targeted proteomics. Drug Metab Dispos 44(12):1920–1924. https://doi.org/10.1124/dmd.116.072066

12. Otsuka M, Matsumoto T, Morimoto R, Arioka S, Omote H, Moriyama Y (2005) A human transporter protein that mediates the final excretion step for toxic organic cations. Proc Natl Acad Sci U S A 102(50):17923–17928

13. Masuda S, Terada T, Yonezawa A, et al (2006) Identification and functional characterization of a new human kidney-specific H+/organic cation antiporter, kidney-specific multidrug and toxin extrusion 2. J Am Soc Nephrol 17(8):2127–2135. https://doi.org/10.1681/ASN.2006030205

14. Prasad B, Evers R, Gupta A, et al (2014) Interindividual variability in hepatic organic anion-transporting polypeptides and P-glycoprotein (ABCB1) protein expression: quantification by liquid chromatography tandem mass spectroscopy and influence of genotype, age, and sex. Drug Metab Dispos 42(1):78–88. https://doi.org/10.1124/dmd.113.053819

15. Wang L, Prasad B, Salphati L, et al (2015) Interspecies variability in expression of hepatobiliary transporters across human, dog, monkey, and rat as determined by quantitative proteomics. Drug Metab Dispos 43(3):367–374. https://doi.org/10.1124/dmd.114.061580

16. Kolesnikov N, Hastings E, Keays M, et al (2015) ArrayExpress update-simplifying data submissions. Nucleic Acids Res 43(D1):D1113–D1116. https://doi.org/10.1093/nar/gku1057

17. Expressed Sequence Tags (EST) from UniGene. National Center for Biotechnology Information (NCBI). https://www.ncbi.nlm.nih.gov/unigene

18. Meyer M, Schneckener S, Ludewig B, Kuepfer L, Lippert J (2012) Using expression data for quantification of active processes in physiologically based pharmacokinetic modeling. Drug Metab Dispos 40(5):892–901. https://doi.org/10.1124/dmd.111.043174

19. Nishimura M, Naito S (2005) Tissue-specific mRNA expression profiles of human ATP-binding cassette and solute carrier transporter superfamilies. Drug Metab Pharmacokinet 20(6):452–477. https://doi.org/10.2133/dmpk.20.452

20. Dallmann R, Brown SA, Gachon F (2014) Chronopharmacology: New insights and therapeutic implications. Annu Rev Pharmacol Toxicol 54(1):339–361. https://doi.org/10.1146/annurev-pharmtox-011613-135923

21. Vaughn B, Rotolo S, Roth H (2014) Circadian rhythm and sleep influences on digestive physiology and disorders. ChronoPhysiology Ther 4:67–77. https://doi.org/10.2147/CPT.S44806

22. Goo RH, Moore JG, Greenberg E, Alazraki NP (1987) Circadian variation in gastric emptying of meals in humans. Gastroenterology 93(3):515–518. https://doi.org/10.1016/0016-5085(87)90913-9

23. Kumar D, Wingate D, Ruckebusch Y (1986) Circadian variation in the propagation velocity of the migrating motor complex. Gastroenterology 91(4):926–930. https://doi.org/10.1016/0016-5085(86)90696-7

24. Lemmer B, Nold G (1991) Circadian changes in estimated hepatic blood flow in healthy subjects. Br J

Clin Pharmacol 32(5):627–629. https://doi.org/10.1111/j.1365-2125.1991.tb03964.x
25. Wesson LG (1964) Electrolyte excretion in relation to diurnal cycles of renal function. Medicine (Baltimore) 43(5):547–592. https://doi.org/10.1097/00005792-196409000-00002
26. Koopman MG, Koomen GCM, Krediet RT, de Moor EA, Hoek FJ, Arisz L (1989) Circadian rhythm of glomerular filtration rate in normal individuals. Clin Sci 77(1):105–111. https://doi.org/10.1042/cs0770105
27. van Acker BAC, Koomen GCM, Koopman MG, Krediet RT, Arisz L (1992) Discrepancy between circadian rhythms of inulin and creatinine clearance. J Lab Clin Med 120(3):400–410
28. Wishart DS, Knox C, Guo AC, et al (2006) DrugBank: a comprehensive resource for in silico drug discovery and exploration. Nucleic Acids Res 34(Database issue):D668-D672. https://doi.org/10.1093/nar/gkj067
29. Desai D, Wong B, Huang Y, et al (2014) Surfactant-mediated dissolution of metformin hydrochloride tablets: Wetting effects versus ion pairs diffusivity. J Pharm Sci 103(3):920–926. https://doi.org/10.1002/jps.23852
30. Graham GG, Punt J, Arora M, et al (2011) Clinical pharmacokinetics of metformin. Clin Pharmacokinet 50(2):81–98. https://doi.org/10.2165/11534750-000000000-00000
31. Tucker GT, Casey C, Phillips PJ, Connor H, Ward JD, Woods HF (1981) Metformin kinetics in healthy subjects and in patients with diabetes mellitus. Br J Clin Pharmacol 12(2):235–246. https://doi.org/10.1111/j.1365-2125.1981.tb01206.x
32. Pentikäinen PJ, Neuvonen PJ, Penttilä A (1979) Pharmacokinetics of metformin after intravenous and oral administration to man. Eur J Clin Pharmacol 16(3):195–202. https://doi.org/10.1007/BF00562061
33. Sirtori CR, Franceschini G, Galli-Kienle M, et al (1978) Disposition of metformin (N,N-dimethylbiguanide) in man. Clin Pharmacol Ther 24(6):683–693. https://doi.org/10.1002/cpt1978246683
34. Yin J, Duan H, Wang J (2016) Impact of substrate-dependent inhibition on renal organic cation transporters hOCT2 and hMATE1/2-K-mediated drug transport and intracellular accumulation. J Pharmacol Exp Ther 359(3):401–410. https://doi.org/10.1124/jpet.116.236158
35. Chen Y, Li S, Brown C, et al (2009) Effect of genetic variation in the organic cation transporter 2 on the renal elimination of metformin. Pharmacogenet Genomics 19(7):497–504. https://doi.org/10.1097/FPC.0b013e32832cc7e9
36. Zhou M, Xia L, Wang J (2007) Metformin transport by a newly cloned proton-stimulated organic cation transporter (plasma membrane monoamine transporter) expressed in human intestine. Drug Metab Dispos 35(10):1956–1962. https://doi.org/10.1124/dmd.107.015495
37. Willmann S, Lippert J, Schmitt W (2005) From physicochemistry to absorption and distribution: predictive mechanistic modelling and computational tools. Expert Opin Drug Metab Toxicol 1(1):159–168. https://doi.org/10.1517/17425255.1.1.159

38. Open Systems Pharmacology Suite Community. Open Systems Pharmacology Documentation. https://docs.open-systems-pharmacology.org/working-with-pk-sim/pk-sim-documentation. Accessed 22 Jan 2022
39. Block LC, Schemling LO, Couto AG, Mourão SC, Bresolin TMB (2008) Pharmaceutical equivalence of metformin tablets with various binders. Rev Ciências Farm Básica e Apl 29(1):29–35
40. Sambol NC, Brookes LG, Chiang J, et al (1996) Food intake and dosage level, but not tablet vs solution dosage form, affect the absorption of metformin HCl in man. Br J Clin Pharmacol 42(4):510–512. https://doi.org/10.1111/j.1365-2125.1996.tb00017.x
41. Buse JB, DeFronzo RA, Rosenstock J, et al (2015) The primary glucose-lowering effect of metformin resides in the gut, not the circulation. Results from short-term pharmacokinetic and 12-week dose-ranging studies. Diabetes Care 39(2):198–205. https://doi.org/10.2337/dc15-0488
42. Idkaidek N, Arafat T (2011) Metformin IR versus XR pharmacokinetics in humans. J Bioequiv Availab 03(10):233–235. https://doi.org/10.4172/jbb.1000092
